# Supplementary material for: Adult Human Dermal Progenitor Cell Transplantation Modulates the Functional Outcome of Split-Thickness Skin Xenografts
Source: Stem Cell Reports. 2019 Nov 14;13(6):1068–82. doi: 10.1016/j.stemcr.2019.10.011 (PMC6915850; doi:10.1016/j.stemcr.2019.10.011)
Supplement: Document S1. Figures S1–S4 and Tables S1–S4 [file mmc1.pdf]

Stem Cell Reports, Volume *13*

Supplemental Information

Adult Human Dermal Progenitor Cell Transplantation Modulates the Functional Outcome of Split-Thickness Skin Xenografts

Natacha A. Agabalyan, Holly D. Sparks, Samar Tarraf, Nicole L. Rosin, Katie Anker, Grace Yoon, Lindsay N. Burnett, Duncan Nickerson, Elena S. Di Martino, Vincent A. Gabriel, and Jeff Biernaskie

SUPPLEMENTAL INFORMATION

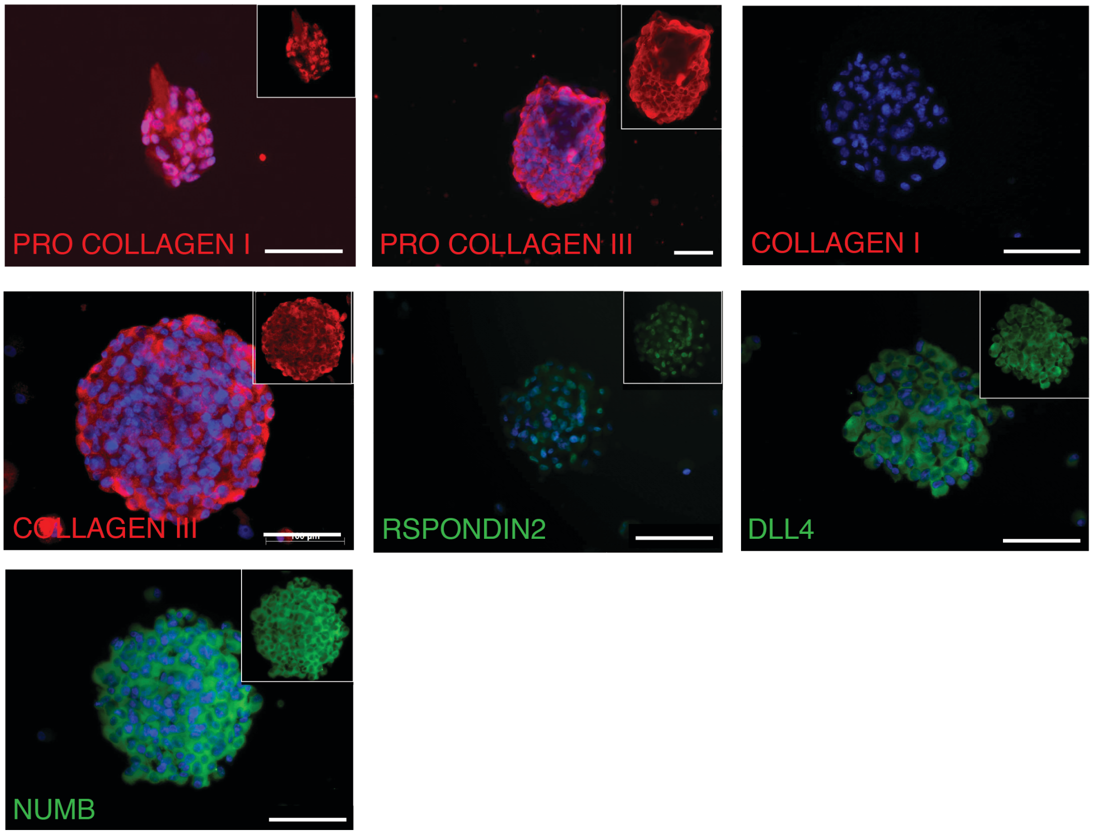

Figure S1. Extended immunocytochemical characterization of hDPCs *in vitro*. Several extracellular matrix proteins were expressed *in vitro*, including PRO COLLAGEN I and III and full-length COLLAGEN III, while full length COLLAGEN I was not. hDPCs in culture were found to consistently express DLL4, NUMB and RSPONDIN2. Scale bar = 100μm.

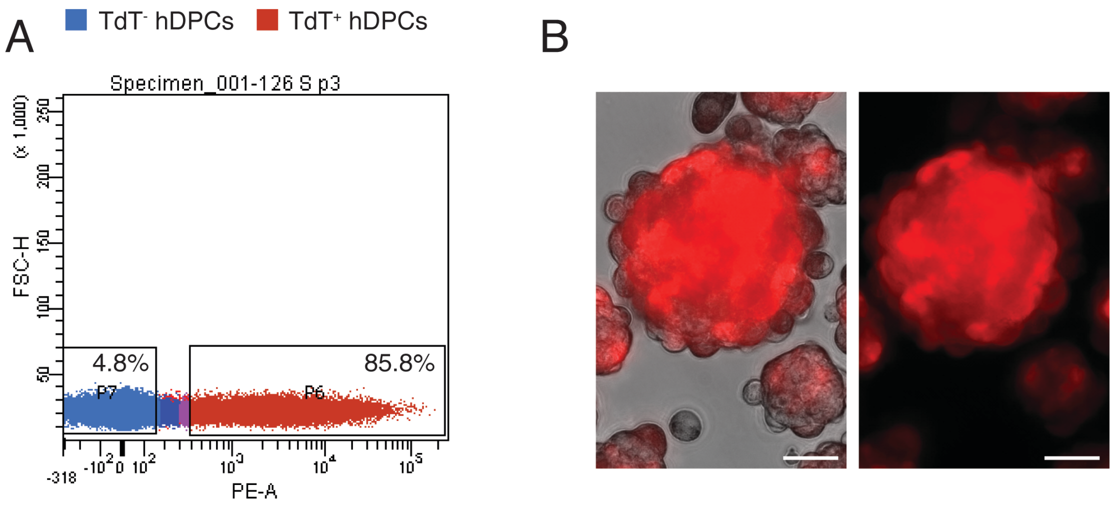

Figure S2. Transfection and FACS of hDPC cells with a TdTomato lentivirus (A) Fluorescence activated cell sorting strategy of TdT+ hDPCs. (B) Images of purified TdT+ hDPC culture following FACS. Scale bar = 100μm.

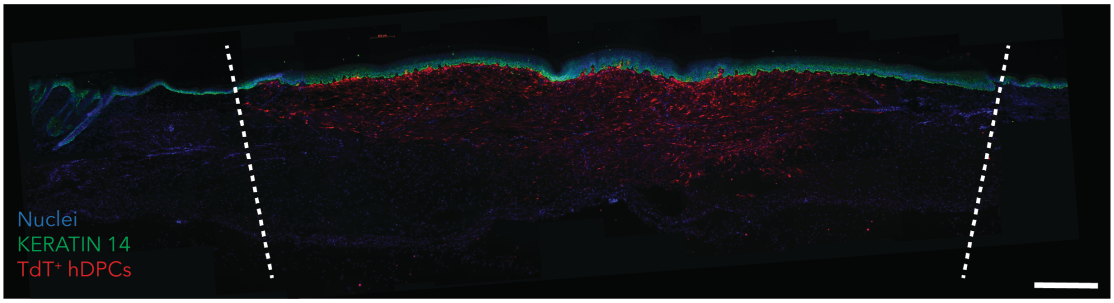

Figure S3. Transplanted TdT+ hDPCs respect the boundaries of the grafted area. Image shows engraftment of TdT+ hDPCs at 3 months *in vivo*. Dashed lines depict the boundaries of the xenograft, with intact mouse skin neighbouring. Labelled cells are not observed outside of the grafted area. Scale bar = 1cm.

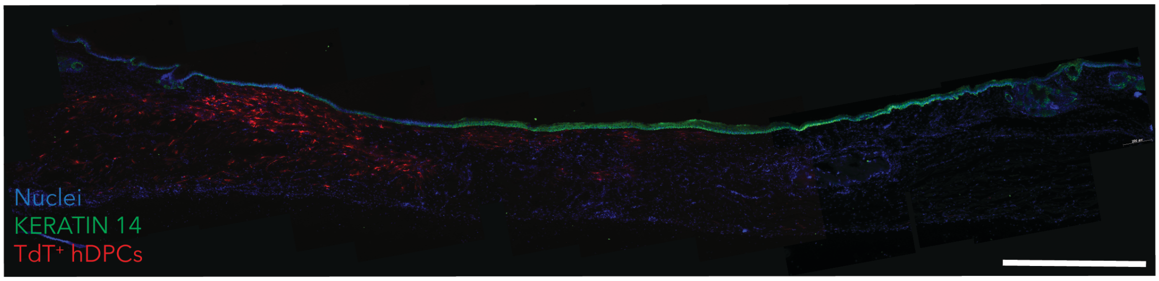

Figure S4. Transplanted TdT+ hDPCs were maintained in the graft for 6 months following transplantation. Image shows engraftment of TdT+ hDPCs *in vivo*. Scale bar = 1cm.

Table S1. Timing of transplants and harvests, clinical characteristics of skin sources

Human skin samples				
	Gender	Age	Anatomical location	Storage prior to harvest
**STSG #57**	Female	52 years old	Abdomen	-80°C in RPMI – thawed just prior to harvest
**STSG #58**	Female	52 years old	Abdomen	-80°C in RPMI – thawed just prior to harvest
**STSG #59**	Female	52 years old	Abdomen	-80°C in RPMI – thawed just prior to harvest
**STSG #60**	Female	N/A	Abdomen	Stored overnight in RPMI media at 4°C prior to harvest
**STSG #61**	Female	53 years old	Abdomen	Stored overnight in RPMI media at 4°C prior to harvest
**STSG #62**	Female	55 years old	Abdomen	Stored overnight in RPMI media at 4°C prior to harvest
**STSG #63**	Female	56 years old	Abdomen	Stored overnight in RPMI media at 4°C prior to harvest
**STSG #64**	Female	55 years old	Abdomen	Stored overnight in RPMI media at 4°C prior to harvest
**STSG #67**	Female	39 years old	Abdomen	-80°C in RPMI – thawed just prior to harvest
**STSG #70**	Female	36 years old	Abdomen	Stored overnight in RPMI media at 4°C prior to harvest
**STSG #71**	Female	36 years old	Abdomen	Stored overnight in RPMI media at 4°C prior to harvest
**STSG #72**	Female	55 years old	Abdomen	Stored overnight in RPMI media at 4°C prior to harvest
**STSG #153**	Female	34 years old	Abdomen	Stored overnight in RPMI media at 4°C prior to harvest

Table S2. Clinical characteristics of cell sources

<table>
<tr><th colspan="5">Human DPC cell sources</th></tr>
<tr><td></td><td>Gender</td><td>Age</td><td>Anatomical location</td><td>Passage</td></tr>
<tr><td>SATP #72</td><td>Male</td><td>46 years old</td><td>Leg</td><td>P7, P8</td></tr>
<tr><td>SATP #65</td><td>Male</td><td>34 years old</td><td>Scalp</td><td>P5, P6</td></tr>
<tr><td>SATP #82</td><td>Female</td><td>78 years old</td><td>Scalp</td><td>P5</td></tr>
<tr><td>SATP #90</td><td>Female</td><td>53 years old</td><td>Scalp</td><td>P7</td></tr>
<tr><td>SATP #122</td><td>Female</td><td>46 years old</td><td>Scalp</td><td>P3</td></tr>
<tr><td>SATP #126</td><td>Male</td><td>66 years old</td><td>Scalp</td><td>P6, P7</td></tr>
<tr><td>SATP #141</td><td>Male</td><td>60 years old</td><td>Scalp</td><td>P6, P9</td></tr>
<tr><th colspan="5">Human Fibroblast cell sources</th></tr>
<tr><td></td><td>Gender</td><td>Age</td><td>Anatomical location</td><td>Passage</td></tr>
<tr><td>SATP #76</td><td>Male</td><td>55 years old</td><td>Leg</td><td>P5</td></tr>
<tr><td>SATP #90</td><td>Female</td><td>53 years old</td><td>Scalp</td><td>P4</td></tr>
<tr><td>SATP #122</td><td>Female</td><td>46 years old</td><td>Leg</td><td>P8</td></tr>
<tr><td>SATP #141</td><td>Male</td><td>60 years old</td><td>Scalp</td><td>P10</td></tr>
<tr><td>SATP #159</td><td>Female</td><td>58 years old</td><td>Scalp</td><td>P6</td></tr>
</table>

Table S3. Primary antibodies used for immunocytochemistry and immunohistochemistry

Antigen	Supplier	Reference	Host	Concentration
**Collagen III**	Rockland	600-401-105S	Rabbit	1:100
**Fgf9**	Millipore	ABN41	Rabbit	1:100
**Fibronectin**	BD Biosciences	610078	Mouse	1:100
**Runx1-3**	Abcam	Ab92336	Mouse	1:50
**Sox2**	Stemgent	O9-0024	Rabbit	1:100
**PDGFR-α**	Santa Cruz	sc-338	Rabbit	1:100
**α-SMA**	Millipore	MABT381	Rabbit	1:100
**FSP1**	Millipore	07-2274	Rabbit	1:100
**Versican**	Millipore	AB1033	Rabbit	1:50
**Grem2**	Abcam	AB189387	Rabbit	1:200
**Pro Collagen I**	Millipore	ABT257	Rabbit	1:100
**Pro Collagen III**	Millipore	AB764P	Rabbit	1:100
**Biglycan**	Santa Cruz	sc-33788	Rabbit	1:100
**Notch1R**	Abcam	AB65297	Rabbit	1:200
**MCAM**	Millipore	04-1147	Rabbit	1:200
**Sox9**	Santa Cruz	sc-166505	Mouse	1:200
**Dll4**	Abcam	AB7280	Rabbit	1:200
**DLK1**	Abcam	AB21682	Rabbit	1:200
**Numb**	Abcam	AB14140	Rabbit	1:500
**Pax1**	ThermoFisher	PA5-51033	Rabbit	1:500
**Rspondin2**	R&D Systems	AF3266	Goat	1:500
**Loricrin**	Covance	PRB-145P	Rabbit	1:500
**Nestin**	Millipore	MAB5326	Mouse	1:100
**Collagen I**	Abcam	ab90395	Mouse	1:200
**Human Nuclear Antigen**	Millipore	MAB1281	Mouse	1:200
**Keratin 14**	Covance	PRB-155P	Rabbit	1:1000
**Ki67**	eBioscience	SolA15	Rat	1:100
**PGP9.5**	Thermofisher	PA5-29012	Rabbit	1:1000

Table S4. Secondary antibodies used for immunocytochemistry and immunohistochemistry

Antigen	Supplier	Reference	Host	Concentration
**Alexa-488**	Invitrogen	A-21206	Donkey anti-rabbit	1:500
**Alexa-488**	Invitrogen	SA1-72000	Donkey anti-chicken	1:500
**Alexa-555**	Invitrogen	A-31570	Donkey anti-mouse	1:500
**Alexa-555**	Invitrogen	A-21432	Donkey anti-goat	1:500
**Alexa-555**	Invitrogen	A-31572	Donkey anti-rabbit	1:500
**Alexa-555**	Invitrogen	A-11008	Goat anti-rabbit	1:500
**Alexa-546**	Invitrogen	A-21098	Donkey anti-sheep	1:500
**Alexa-647**	Invitrogen	A-21245	Goat anti-rabbit	1:500
**Alexa-647**	Invitrogen	A-21447	Donkey anti-goat	1:500
**Alexa-647**	Abcam	ab150155	Donkey anti-rat	1:500
**Hoechst**	Sigma-Aldrich	14530		1:1000
