# Supplementary material for: Adult Human Dermal Progenitor Cell Transplantation Modulates the Functional Outcome of Split-Thickness Skin Xenografts
Source: Stem Cell Reports. 2019 Nov 14;13(6):1068–82. doi: 10.1016/j.stemcr.2019.10.011 (PMC6915850; doi:10.1016/j.stemcr.2019.10.011)
Supplement: Document S2. Article plus Supplemental Information [file mmc2.pdf]

Stem Cell Reports
Article
OPEN ACCESS

Adult Human Dermal Progenitor Cell Transplantation Modulates the Functional Outcome of Split-Thickness Skin Xenografts

Natacha A. Agabalyan,[1,10] Holly D. Sparks,[1,10] Samar Tarraf,[4] Nicole L. Rosin,[1] Katie Anker,[1] Grace Yoon,[1] Lindsay N. Burnett,[2] Duncan Nickerson,[2,3] Elena S. Di Martino,[4,9] Vincent A. Gabriel,[2,5,6,7] and Jeff Biernaskie[1,3,7,8,*]

[1]Department of Comparative Biology and Experimental Medicine, Faculty of Veterinary Medicine, University of Calgary, Calgary, AB, Canada
[2]Calgary Firefighters Burn Treatment Centre, Calgary, AB, Canada
[3]Section of Plastic Surgery, Department of Surgery, University of Calgary, Calgary, AB, Canada
[4]Biomedical Engineering Graduate Program, University of Calgary, Calgary, AB, Canada
[5]Departments of Clinical Neurosciences, Surgery and Paediatrics, University of Calgary, Calgary, AB, Canada
[6]McCaig Institute of Bone and Joint Research, Cummings School of Medicine, University of Calgary, Calgary, AB, Canada
[7]Alberta Children's Hospital Research Institute, Calgary, AB, Canada
[8]Hotchkiss Brain Institute, Calgary, AB, Canada
[9]Department of Civil Engineering, Centre for Bioengineering Research and Education, University of Calgary, Calgary, AB, Canada
[10]Co-first author
*Correspondence: jeff.biernaskie@ucalgary.ca
https://doi.org/10.1016/j.stemcr.2019.10.011

SUMMARY

Following full-thickness skin injuries, epithelialization of the wound is essential. The standard of care to achieve this wound "closure" in patients is autologous split-thickness skin grafting (STSG). However, patients living with STSGs report significant chronic impairments leading to functional deficiencies such as itch, altered sensation, fragility, hypertrophic scarring, and contractures. These features are attributable to the absence of functional dermis combined with the formation of disorganized fibrotic extracellular matrix. Recent work has demonstrated the existence of dermal progenitor cells (DPCs) residing within hair follicles that function to continuously regenerate mesenchymal tissue. The present work examines whether cultured DPCs could regenerate dermis within an STSG and improve overall graft function. Adult human DPCs were transplanted into a full-thickness skin wound in immune-compromised mice and closed with a human STSG. At 3 months, human DPCs (hDPCs) had successfully integrated into the xenograft and differentiated into various regionally specified phenotypes, improving both viscoelastic properties of the graft and mitigating pruritus.

INTRODUCTION

Superficial wounds heal well and without the need for surgical interventions. However, in the setting of a deep insult to the skin that destroys (or requires the removal of) most of the dermis, patients do not have the capacity to heal with functional tissue. Thus, the current standard of care following full-thickness (FT) skin injury such as in severe burns, trauma, complicated soft tissue infections, or surgical resections is split-thickness skin grafting (STSG) (American Burn Association, 2016). In this procedure, the epidermis and a thin portion of the superficial dermis is harvested from an uninjured area of the patient's body and applied to the open wound after it has been adequately prepared through the removal of necrotic, neoplastic, or infected tissue. This provides effective wound closure, restoration of barrier function, and improved mortality rates among critically injured patients (Ong et al., 2006). However, the grafted skin typically remains chronically dysfunctional. These patients experience chronic pruritus, altered sensation, fragility, hypertrophic scarring, and contractures that may lead to reduced range of motion and ultimately impairments and disability (Burnett et al., 2014).

This scar formation and consequent dysfunction are due to the absence of a complete functional dermal tissue that is not harvested from the donor site in STSG. In healthy intact skin, the dermis provides critical structural and trophic support to the epidermis (Crandall and Davis, 2010), as well as housing components of functional cutaneous appendages, such as hair follicles, nerves, blood vessels, and glands. Although various forms of skin grafting techniques have evolved over time, the current surgical procedures have not changed significantly since the adoption of tangential excision and grafting in the 1970s (Lee et al., 2014). Attempts have been made to improve the appearance of the resultant scar following STSG through the application of an acellular dermal matrix under an STSG in human patients (Yi and Kim, 2015). Engineered bilayer skin "substitutes" in which cultured sheets of human epithelial cells are combined with dermal fibroblasts have also shown similarly improved outcomes, highlighting the importance of neodermis to restoration of skin function (Auger et al., 2004; Cantin-Warren et al., 2018; Gauvin

 © 2019 The Authors.
This is an open access article under the CC BY license (http://creativecommons.org/licenses/by/4.0/).

et al., 2013; Nyame et al., 2015). Despite these advances, function of the grafted area remains a concern for patients (Burnett et al., 2014).

Dermal fibroblasts are functionally diverse and each population lends distinctly different contributions to the wound healing process (Biernaskie et al., 2009; Driskell et al., 2013; Philippeos et al., 2018). Fibroblasts isolated from the upper dermis consistently display evidence of an anti-inflammatory, pro-regenerative phenotype, and the ability to support the epidermis in co-culture, whereas fibroblasts residing in deep layers appear preferentially fibrotic, suggesting the anatomical location (and perhaps developmental origin) of various fibroblast populations might also imply divergent functional and regenerative potential (Ding and Tredget, 2017; Wang et al., 2008).

Our work has demonstrated the existence of a specialized fibroblast, or a self-renewing dermal progenitor cell (DPC), residing at the base of the hair follicle (Biernaskie et al., 2009) that functions to continuously repopulate the mesenchyme and thus enable hair follicle regeneration (Rahmani et al., 2014). These cells can be isolated and expanded *in vitro* as self-renewing spherical colonies (previously referred to as “skin-derived precursors” [SKPs], now as DPCs). When transplanted to FT skin wounds, rodent DPCs proliferate to fill the wound with neodermal tissue and, when combined with competent epithelial keratinocytes, are able to induce *de novo* hair follicles (Biernaskie et al., 2009). Based on their unique inherent regenerative capacity in the absence of fibrosis, we hypothesize that autologous human DPCs (hDPCs) could be isolated from a small biopsy of intact skin, expanded in culture, then transplanted underneath an autologous STSG in an effort to improve neodermal regeneration and functional outcomes following STSG in human patients requiring treatment of severe skin injury.

To begin to investigate this concept, we have developed a human-to-mouse STSG xenograft model using homozygous nude mice. TdTomato (TdT)-labeled hDPCs harvested from adult human scalp were transplanted into a FT skin wound in nude mice and covered by a human STSG. Here, we describe the isolation and characterization of adult hDPCs, the refinement of a xenograft model, and the fate and impact of hDPC transplant within the STSG environment. At 3 months, donor hDPCs successfully integrated into the grafted region and differentiated into various regionally specified phenotypes. Inclusion of a collagen scaffold greatly improved cell distribution and expansion within the graft. However, when an empty collagen scaffold was included, graft take was negatively affected. This reduction in graft viability was mitigated by inclusion of hDPCs. Within the graft, transplanted hDPCs generated neodermis that resulted in increased elasticity and reduced itch. Interestingly, the addition of cultured interfollicular dermal fibroblasts (hFs), under an STSG also showed modest benefit on itch.

RESULTS

Isolated Adult Human DPCs Retain Expression of the Hair Follicle Mesenchyme

Adult DPCs were isolated from donor human scalp skin as described previously (Biernaskie et al., 2007). Over 7–21 days, floating spherical colonies were observed and allowed to grow until approximately 200–300 μm in diameter before being dissociated and passaged (Figure 1A).

Following three to five passages, hDPCs-expressed proteins consistent with a hair follicle mesenchyme origin (Biernaskie et al., 2009; Fernandes et al., 2004; Rahmani et al., 2014) including extracellular matrix (ECM) proteoglycans versican and biglycan, and the transcription factors RUNX, SOX2, and PAX1, all of which are enriched in the rodent hair follicle dermal papilla (Figure 1B). Although both pro-collagen I and pro-collagen III peptides were expressed by hDPCs in culture, only full-length collagen III was present in spheres (Figure S1). Dermal fibroblast markers fibronectin, α-SMA, FSP-1, and PDGFR-α, were also present (Figure 1B). Proteins involved in both Wnt and Notch signaling including NUMB, DLL4, and RSPONDIN 2 were similarly expressed by hDPCs in culture (Figure S1). NESTIN, an intermediate filament protein enriched in mesenchymal cells within the connective tissue sheath (Tiede et al., 2009) that harbors hDPCs in rodents (Biernaskie et al., 2009; Rahmani et al., 2014), was similarly identified in isolated hDPCs (Figure 1B).

Inclusion of a Dermal Scaffold Promotes Robust Engraftment of hDPCs

Fresh, FT abdominal surgical waste tissue from white female patients aged 34–56 years (Table S1) was collected and STSGs were harvested using an electric Padgett dermatome (0.015″ setting). These STSGs were then grafted onto 12 mm diameter, FT excisional back skin wounds on immune-deficient adult nude (Nu/Nu) mice, either alone or atop a US Food and Drug Administration-approved collagen III scaffold (S) (Primatrix, Integra) and with or without TdT+ hDPCs or TdT+ hFs. Grafts were harvested at 1, 2, and 3 months following transplant for immunohistochemical analysis. The cell-sorting strategy and appearance of TdT+ hDPCs following lentiviral transduction can be observed in Figure S2. Transplanted human DPCs successfully integrated into the STSG (Figure 2A) and exhibited sustained integration within the STSG even at 3 months after transplant in many grafts. Transplanted hFs were retained only very rarely following 3 months. Although fewer overall STSG/S/hDPC grafts

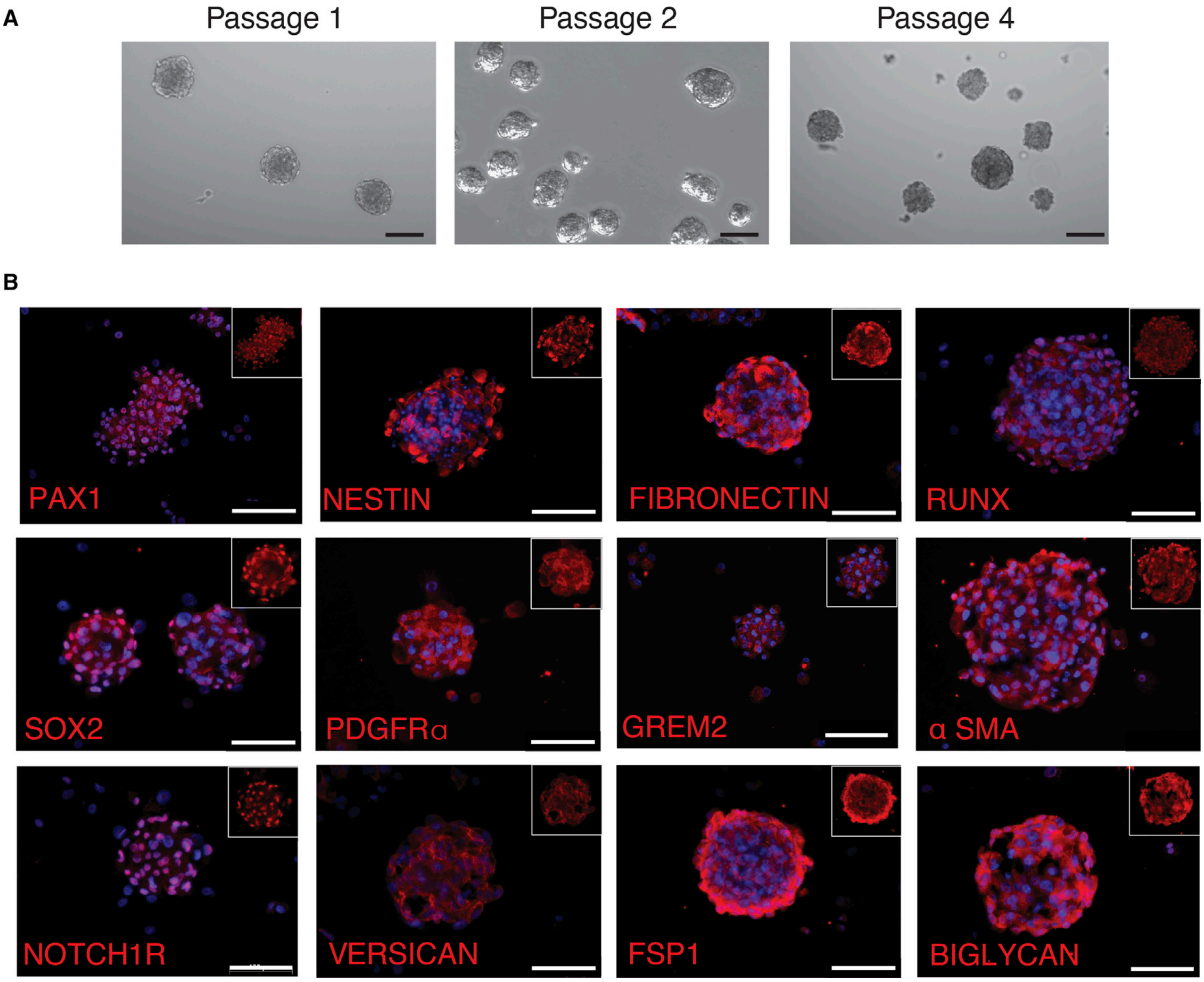

Figure 1. Human Dermal Progenitor Cell (hDPC) Isolation, Expansion, and Characterization
(A) hDPC culture over several passages.
(B) hDPC expression of specific markers including PAX1, NESTIN, FIBRONECTIN, RUNX, SOX2, PDGFR-α, GREM2, α-SMA, NOTCH1R, VERSICAN, FSP1, and BIGLYCAN.
Scale bars, 100 μm.

contained cells at 3 months compared with STSG/hDPC alone (Figure 2B), cell engraftment was more robust in the group seeded on the scaffold (STSG/S/hDPC) as observed by a greater total number of cells retained within the graft (Figures 2A and 2C), suggesting that inclusion of a biophysical scaffold is critical to cell survival and robust formation of neodermis within the graft. Importantly, no cells were found remote to the grafted area (Figure S3) and no evidence of excessive or abnormal accumulation of cells or tissue was observed in any of the transplanted mice (n = 97). Notably, the scaffold allows for greater engraftment and enhanced survival of the transplanted hDPCs, enabling effective formation of neodermis that fills the depth and width of the grafted area (Figure 2D).

Grafts performed with a scaffold showed a significantly more robust filling of the wound area (30%) compared with grafts with cells alone (10%) (Figure 2C). The addition of the scaffold significantly increased the dermal thickness both with and without cells compared with STSG alone, yet all grafted groups remained significantly thinner than that of human FT (hFT) skin. The addition of hDPCs alone significantly reduced dermal thickness compared with the scaffold alone (Figure 2E). Based on the improved cell engraftment observed following use of a scaffold, all further analyses were conducted only utilizing cells delivered atop a scaffold (STSG/S/hDPC, STSG/S/hFs) in an effort to reduce the total number of experimental animals required.

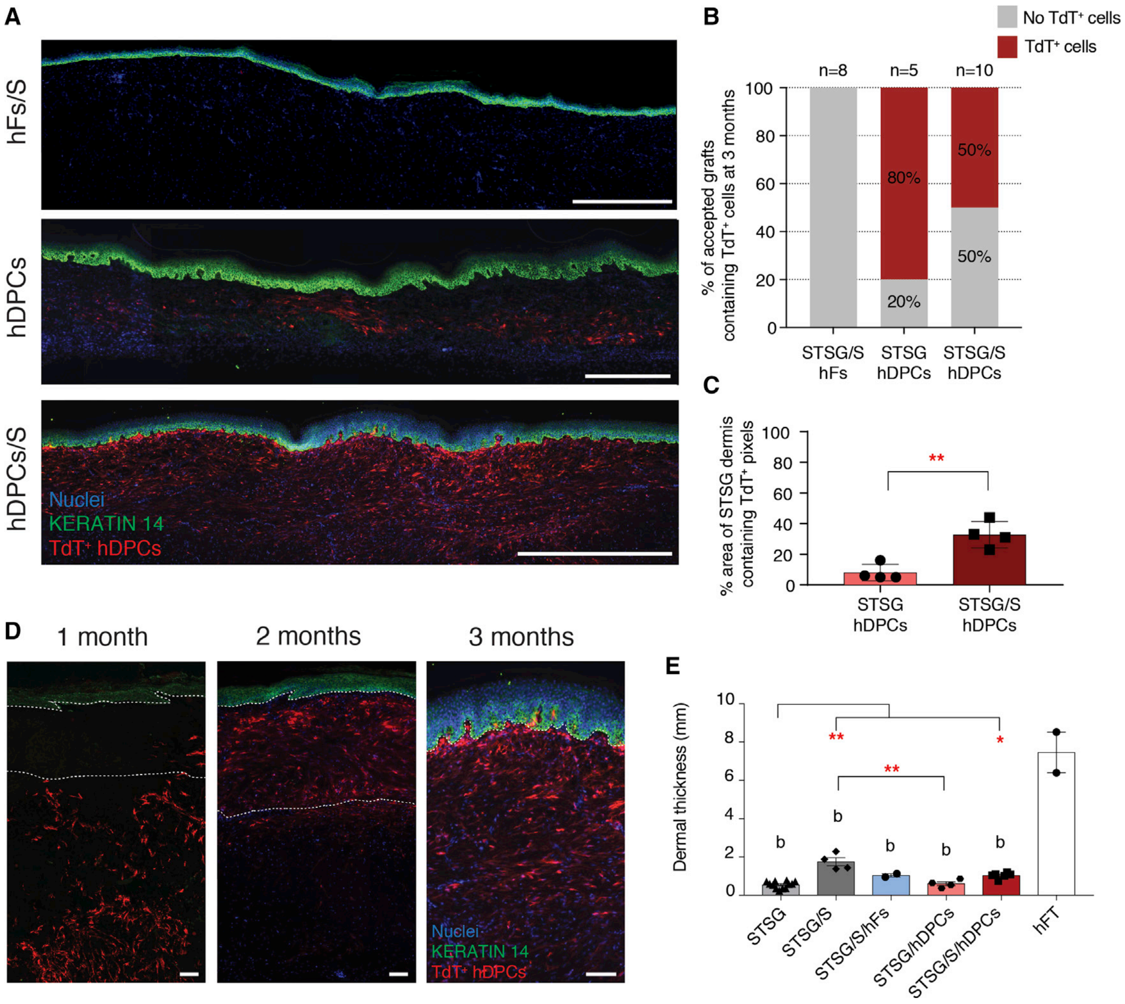

Figure 2. Transplanted TdT+ hDPCs Survive, Engraft, and Distribute across the Wound in the Xenograft Model

(A) Engraftment of TdT+ hDPCs *in vivo* at 3 months. Scale bars, 1 cm.

(B) Quantification of engraftment of TdT+ hDPCs at 3 months.

(C) Quantification of area of graft with TdT+ hDPCs.

(D) Survival and distribution of TdT+ cells throughout the wound bed over the course of 3 months. Dashed line depicts the boundary between the scaffold and STSG. Scale bars, 100 μm.

(E) Quantification of dermal thickness.

Data and error bars in (C) and (E) represent means ± SEM (n = 2–12 mice per group). $^*p < 0.05$, $^{**}p < 0.01$ between treatment groups. (a) $p < 0.05$, (b) $p < 0.01$ compared with hFT.

Viability of Human Split-Thickness Xenografts Is Hindered by the Addition of a Scaffold, but Partially Rescued with Inclusion of Cells

By week 4, successful graft viability, or "take" was observed, with grafted skin being well-integrated into the host wound and confluent with the surrounding uninjured skin. Grafts that were deemed to have good graft take were re-vascularized and pliable (Figure 3A). By 3 months, most grafts showed increased pigmentation relative to the initial appearance of the donor skin (Figure 3B). At 3 months post grafting, the human-mouse split-thickness xenograft (Figure 3C) appeared histologically similar to STSG biopsied from human patients at 5 months post grafting (Figure 3D) containing an intact epidermal layer and unstructured dermal layer beneath. This absence of dermal appendages (e.g., hair follicles, glands) was found, as expected following an STSG.

Graft take was scored by a blinded observer subjectively as either lost, partially lost, or accepted. Grafting outcome was negatively affected by the inclusion of an acellular dermal scaffold, but the addition of hDPCs appeared to

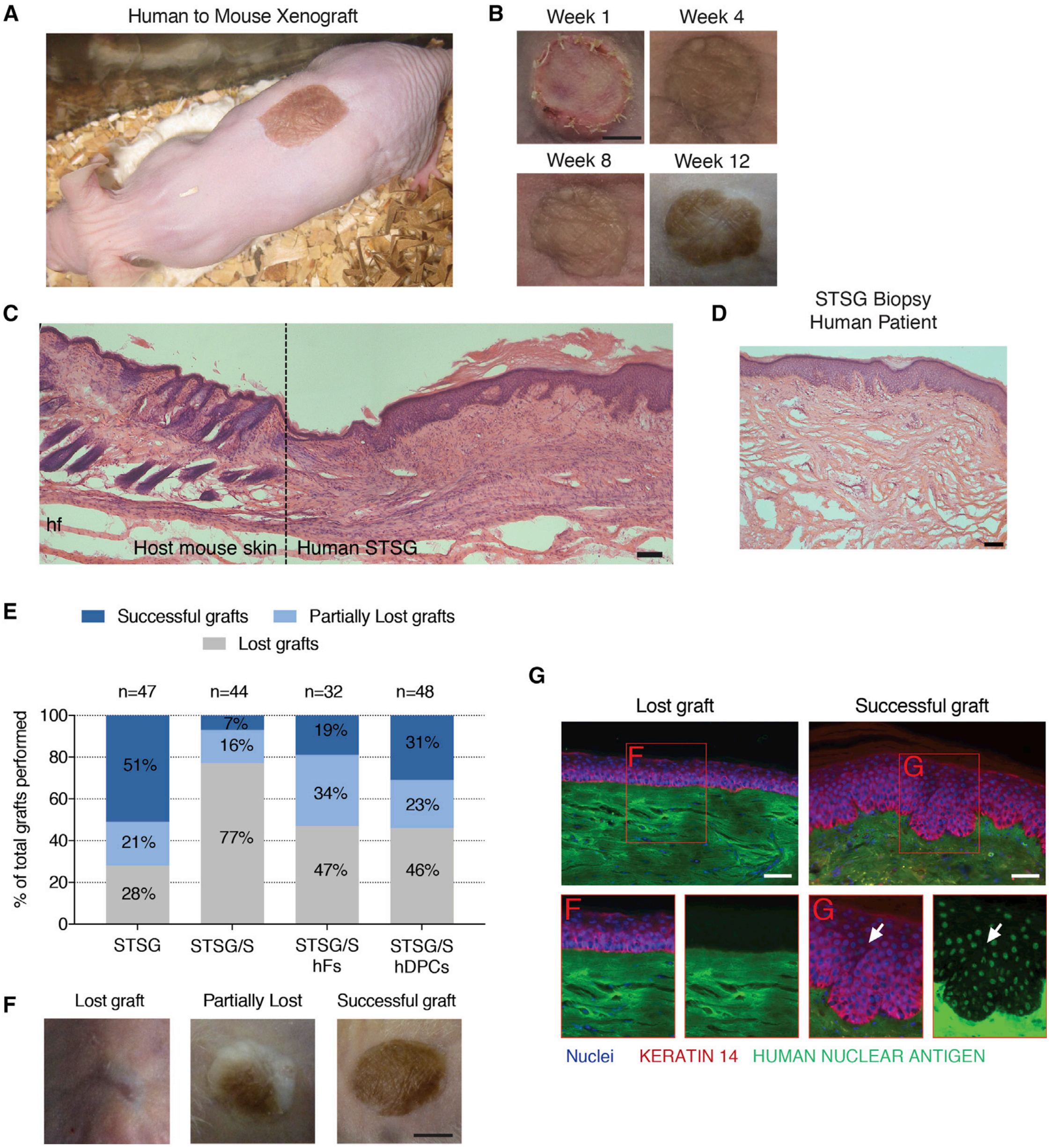

Figure 3. Integration of Human Split-Thickness Skin Xenograft within Recipient Mice
(A) Image of a successful human-to-mouse xenograft 4 weeks post grafting.
(B) Gross images of successful graft take after 1, 4, 8, and 12 weeks. Scale bar, 500 μm.
(C) H&E staining of integration of human STSG into the host mouse skin 3 months post grafting.
(D) Image of a human STSG biopsied 5 months post grafting. Scale bar, 100 μm.
(E) Quantification of graft take at 3 months.
(F) Images of grafts grossly scored as lost, partially lost, or accepted. Scale bar, 500 μm.
(G) Positive human nuclear antigen staining (white arrows) in successful grafts. Scale bars, 100 μm.

rescue this effect (Figures 3E and 3F). STSGs alone had over 70% successful take, but the addition of the empty scaffold reduced this to 23%. The addition of cells (either hDPCs or hFs) appeared to rescue the negative effect of the scaffold with both groups increasing success to 50%.

Immunolabelling with human nuclear antigen confirmed the blinded observer scoring by identifying the presence of human cells comprising the epidermis of the grafted tissue and their absence within grafts that were lost (Figure 3G).

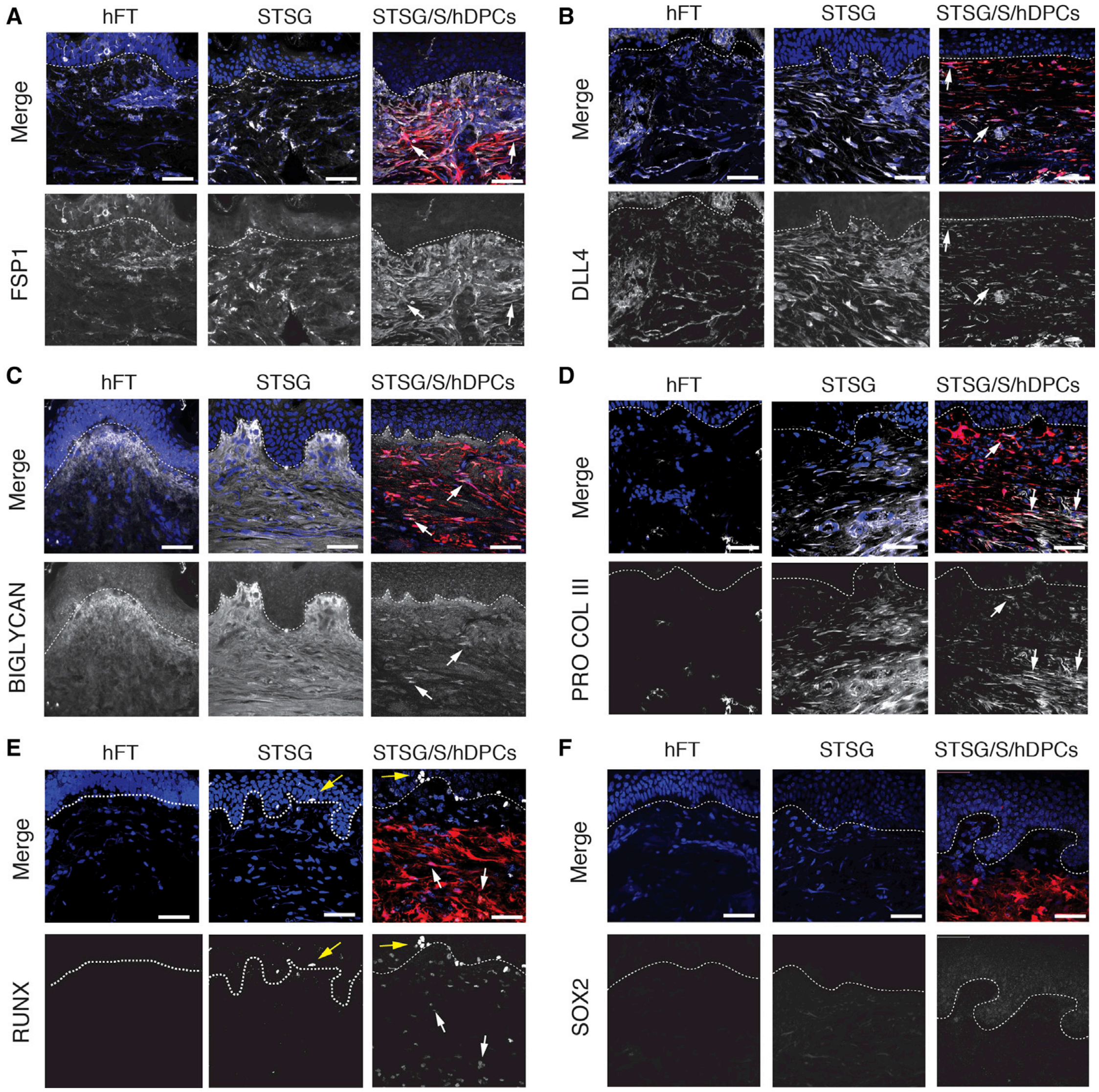

Figure 4. Transplanted hDPCs Successfully Engraft and Differentiate within the STSG

(A) Representative images of FSP1 (A), DLL4 (B), BIGLYCAN (C), PRO-COLLAGEN III (D), RUNX (E), and SOX2 (F) in hFT skin, STSG alone, and STSG/S/hDPCs. Nuclei in blue, TdT+ cells in red, antibodies in gray. Scale bars, 50 μm.

Transplanted hDPCs Acquire Various Phenotypes Dependent on Local Cues within the Graft

To assess the fate of transplanted cells within each graft, we performed immunostaining for various mesenchymal markers at 3 months in grafts supplemented with TdT+ hDPCs as well as control STSG alone and ungrafted (FT) human abdominal skin. As TdT+ hFs were not observed to persist in grafts at 3 months, they were omitted from our immunohistochemical analysis.

Most fibroblast markers expressed by isolated hDPCs *in vitro* were sustained following transplantation including FSP1, DLL4, RUNX1-3, pro-collagen III, and biglycan (Figures 4A–4E). Qualitatively, STSG supplemented by the addition of hDPCs exhibited a diminished level of expression of DLL4 and biglycan compared with STSG only controls. Many TdT+ hDPCs were found to express pro-collagen III (Figure 4); however, when compared with STSGs without cells, a lower overall amount of pro-collagen III was observed following the addition of hDPCs (Figure 4). Importantly, although many markers associated with the hair follicle mesenchyme (such as SOX2 and fibronectin) were diminished in hDPCs residing in the graft (Figure 4F); RUNX was observed in the epidermis of both STSG with and without cells, but only remained active in a subset of

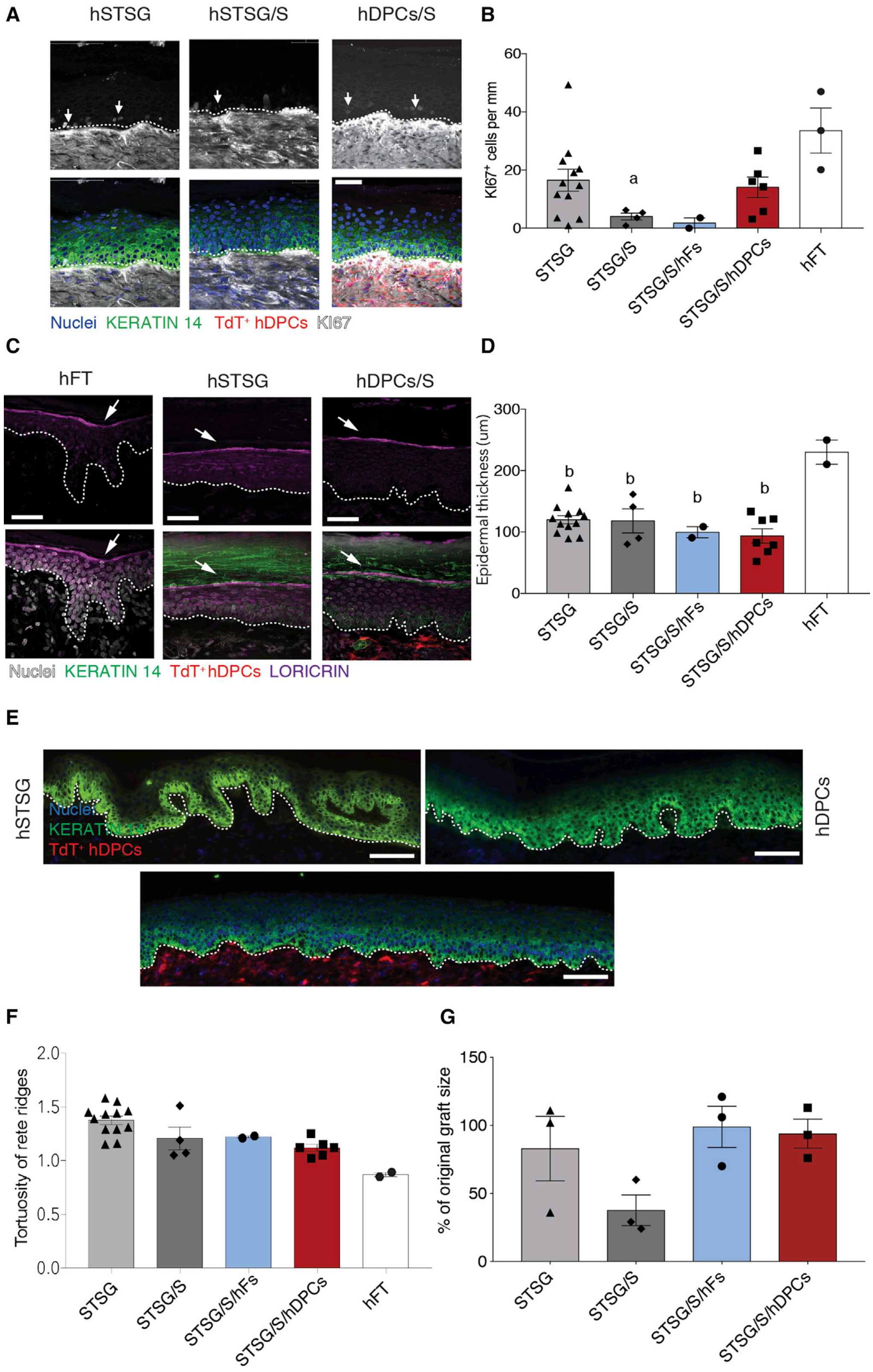

(legend on next page)

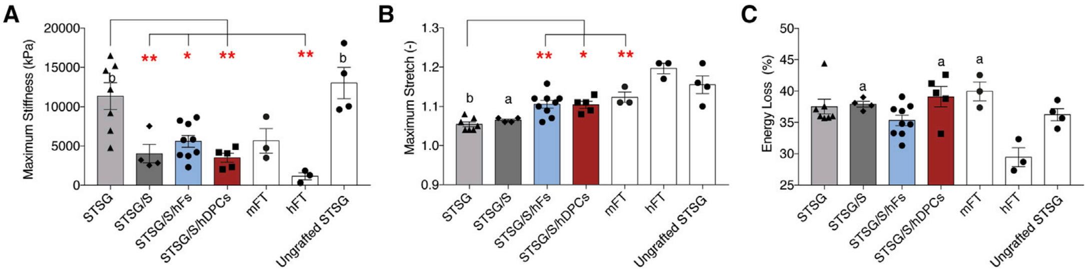

Figure 6. Transplanted hDPCs Improve Xenograft Viscoelasticity

(A) Quantification of maximum stiffness (A), maximum stretch (B), and energy loss (C) generated from the biaxial data for all treatment groups. *$p < 0.05$, **$p < 0.01$ between treatment groups. (a) $p < 0.05$, (b) $p < 0.01$ compared with hFT.

TdT+ cells located beneath the dermal-epidermal interface (Figure 4E), suggesting that differentiation and phenotypic fate of donor cells is influenced by local microenvironmental cues.

Presence of a Scaffold, but Not Cell Transplantation, Alters Epidermal Morphology and Function

The addition of hDPCs affected proliferation of basal layer epidermal progenitor cells in the overlying graft as demonstrated by KI67 staining (Figure 5A). While addition of the scaffold lowered the number of positive cells compared with STSG alone, the addition of hDPCs (but not hFs) appeared to rescue this trend, with an increase toward that of intact skin. Addition of the scaffold alone significantly decreased the number of KI67+ cells compared with hFT skin. Interestingly, many KI67+ cells in all grafted groups were observed, subjectively, to be located in the suprabasal layer rather than exclusive to the basal layer (Figure 5A, arrows). Examination of Loricrin, which marks differentiated epidermis, showed that it was maintained in all accepted STSGs irrespective of treatment (Figure 5C).

Epidermal thickness was not affected by the addition of cells or the scaffold (Figure 5D), but all grafts were found to be significantly thinner than native human abdominal skin. The rete ridges in the STSG alone and STSG/S/hDPCs showed an increase in the tortuosity and depth of rete ridges relative to hFT skin (Figures 5E and 5F). To assess whether graft contraction was a potential source of this increase in tortuosity, the graft size was assessed at 3 months in comparison with the original wound size. Although graft contraction was observed, no significant differences were found between groups (Figure 5G).

Supplementing STSG with Cultured Dermal Cells Improves Mechanical Function and Reduces Pruritus

To assess the impact of the addition of cells on biophysical function of each graft, a subset of (2 × 2cm) grafts were harvested at 3 months and analyzed using a planar biaxial testing system (Figure 6A). Relative to STSG alone, STSG/S or STSG/S/hDPCs and STSG/S/hFs, exhibited a significant reduction in maximum stiffness, becoming more similar to that of hFT skin. Only STSG alone and freshly harvested, ungrafted STSG were observed to have significantly increased stiffness compared with hFT. Both STSG/S/hDPCs and STSG/S/hFs significantly increased maximum stretch compared with STSG alone, and again more closely resembled hFT skin (Figure 6B). Only STSG alone and STSG/S showed significantly diminished stretch compared with hFT. STSG/S, STSG/S/hDPCs, and FT mouse skin were found to have significantly increased energy loss levels compared with hFT skin (Figure 6C).

To assess the frequency and severity of pruritus we evaluated the incidence of scratch marks on or near the graft site (Figure 7A) and scored them as either none, moderate, or severe. Addition of the scaffold alone increased the incidence of severe scratching from 8% (STSG alone) to 25%. In contrast, STSG/S/hDPCs and STSG/S/hFs showed no instances of severe scratching (Figure 7B). Similarly,

Figure 5. Impact of Scaffold and Cell Transplant on Epidermal Function

(A) Representative image of KI67 (white, white arrow) labeling in the grafted epidermis.
(B) Analysis of KI67+ nuclei per mm in the grafted epidermis.
(C) Representative image of LORICRIN (mauve, white arrow) expression in the grafted epidermis.
(D) Quantification of epidermal thickness in the grafted epidermis.
(E) Images of epidermal rete ridges following grafting. Scale bars, 100 μm.
(F) Quantification of tortuosity of rete ridges in the grafted epidermal layer.
(G) Quantification of graft contraction 3 months post grafting.
Data and error bars represent means ± SEM (n = 2–12 mice per group). *$p < 0.05$ between treatment groups. (a) $p < 0.05$, (b) $p < 0.01$ compared with hFT.

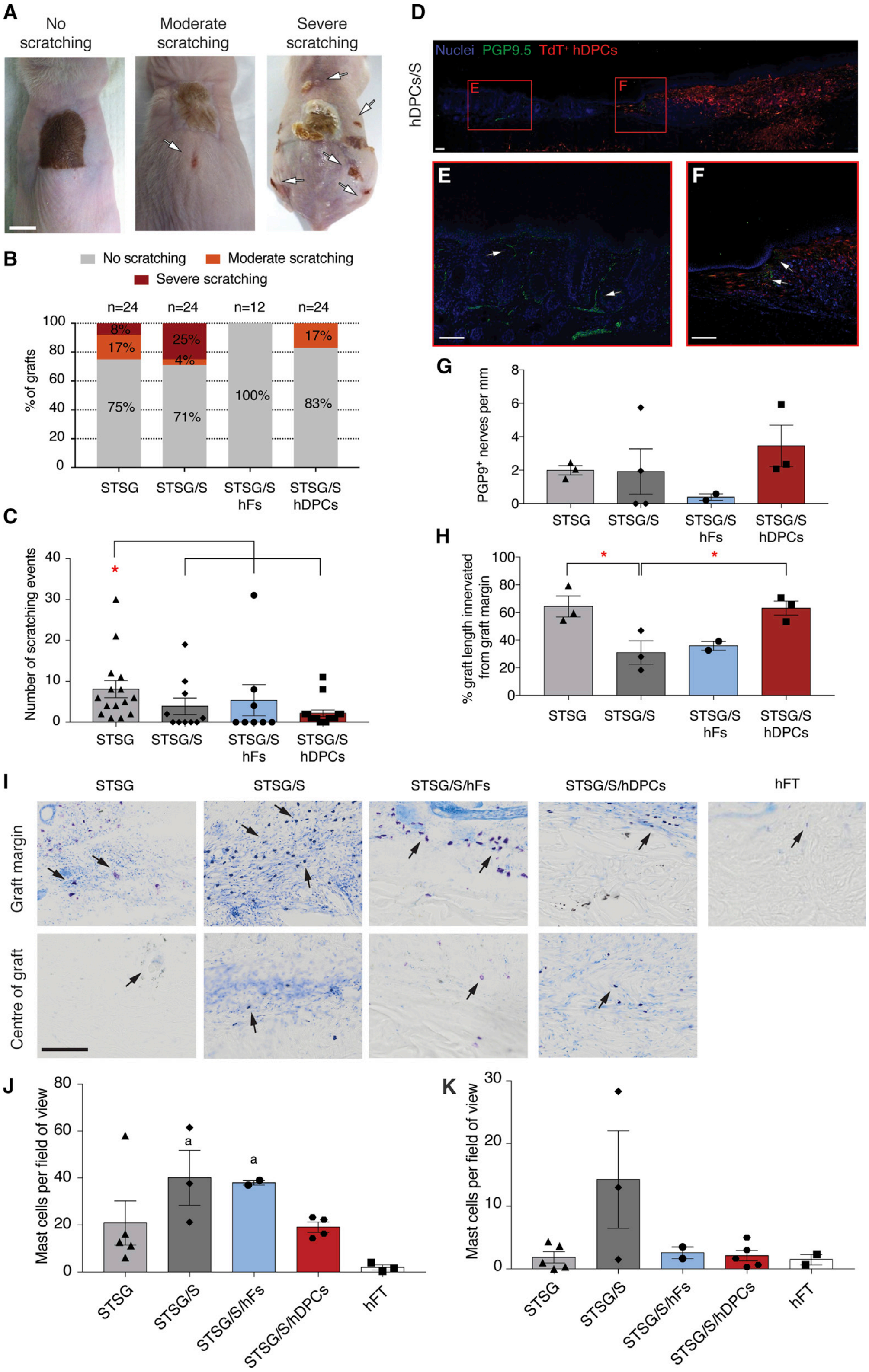

(legend on next page)

behavioral quantification of the number of scratching events observed was significantly decreased in all treatment groups compared with STSG alone (Figure 7C). However, it is important to note that the magnitude of improvement in the hDPC- and hF-treated groups is grossly underestimated as many of the animals that exhibited severe scratching/pruritus (particularly in STSG alone and STSG/S groups) were removed from the study early based on exceeding our humane endpoints and so were not included in the scratching frequency behavioral quantification (Figure 7C).

To better appreciate the potential mechanism by which cells may be influencing pruritus within the graft, three sets of STSG were analyzed for the presence of re-innervation (as indicated by PGP9.5+ nerve fibers) and mast cells. Immunohistochemistry revealed evidence of small nerve fibers increasing in density toward TdT+ hDPCs in the graft site (Figures 7D–7F). Quantification of nerve fibers crossing the basement membrane into the human STSG epidermis revealed no differences in density of nerve fibers across treatment groups (Figure 7G); however, addition of the scaffold showed a significant decrease in the percentage of graft length innervated with PGP9.5+ fibers compared with STSG alone or STSG/S/hDPCs (Figure 7H).

Finally, since mast cells have been implicated in the pathogenesis of itch as well as contracture (Monument et al., 2015), we quantified their frequency within different regions of each graft. Toluene blue staining (Figure 7I) revealed a significant increase in density of mast cells at the margin of the graft (Figure 7J) and at the center of the graft (Figure 7K) in STSG/S compared with hFT skin. However, addition of hDPCs did not result in elevated mast cell presence, suggesting that the addition of DPCs may modulate persistence or activation of mast cells in the graft that may contribute to chronic itch.

DISCUSSION

This work is of potentially significant clinical importance as the application of an STSG is the current standard of care for FT skin injuries. This procedure is largely successful and has been instrumental in reduction of burn-related mortality in regions with adequate infrastructure. Despite this, chronic functional deficiencies of the STSG pose lifelong challenges for patients (Burnett et al., 2014). Incorporating autologous hDPCs along with autologous STSG, represents a unique strategy to improve STSG outcome as demonstrated here by improving two key patient-identified STSG morbidities, namely graft stiffness/fragility and chronic itch. Regarding the feasibility of this approach, the vast majority of FT skin injuries in North America are of a relatively small total body surface area (TBSA), whereas those of massive FT TBSA injuries (such as burns), that do not have adequate uninjured skin to harvest enough STSG, are rare (Smolle et al., 2016; American Burn Association, 2016). Hence, excessive numbers of cells and long periods of expansion would not be required for most procedures. As well, most common small TBSA burns are not excised and grafted in the first hours post injury, but instead are planned often several days in advance. Outpatient surgery along with shorter hospital stays for admitted patients are becoming more common (Abdelrahman et al., 2017; Engrav et al., 2013). Thus, the time required for cell isolation and *in vitro* expansion would be feasible in such cases.

In this work, we describe the successful isolation of DPCs from adult human skin and their use for dermal regeneration in a xenograft model of STSG. There were several important findings from this study. First, adult human DPCs can indeed be isolated, expanded *in vitro*, and subsequently incorporated into an STSG where they actively migrate to repopulate the wound site, interact with the graft and produce new dermal tissue that persists for at least 3 months after transplant. Transplanted hFs (despite having some functional benefit) showed a marked decline in frequency within the graft, to almost undetectable levels by 3 months. Second, hDPC integration and distribution across the graft was improved when cells were delivered on a scaffold. However, the presence of the selected collagen scaffold was not without consequence. In fact, the presence of the scaffold alone, without incorporation

Figure 7. Supplementing STSG with Dermal Cells Limits Pruritus

(A) Images of moderate and severe scratching in mice after 3 months. Scale bar, 1 mm.

(B) Scoring of scratching at 3 months.

(C) Quantification of the number of scratching events at 3 months.

(D–F) (D) Representative image of PGP9.5 staining at the graft margin (E) and within the wound bed (F) (green, arrows) Scale bars, 200 μm.

(G) Quantification of number of PGP9.5+ fibers crossing the basement membrane into the epidermis.

(H) Quantification of the percentage of graft innervated by PGP9.5+ fibers.

(I) Representative images of toluene blue staining. Scale bar, 100 μm.

(J) Quantification of mast cells at the graft margin.

(K) Quantification of mast cells in the center of the graft.

Data and error bars represent means ± SEM (n = 2–5 mice per group). *$p < 0.05$, **$p < 0.01$ between treatment groups. (a) $p < 0.05$, (b) $p < 0.01$ compared with hFT.

of cells, increased levels of pruritus and was associated with a dramatic reduction in graft take. This could be mediated by the addition of either hDPCs or hFs into the scaffold. Most importantly, supplementing STSG with hDPCs (and to a lesser extent, hFs) resulted in the reduction of pruritus and improvement of biomechanical function of STSG relative to STSG alone.

Following successful isolation of DPCs from adult human scalp and leg skin, characterization of the cells revealed maintenance of mesenchymal stem cell markers as well as expression of proteins involved in human hair follicle maintenance, similar to the expression profile of rodent DPCs. Although most of the isolated cells showed a consistent expression pattern, several markers (including SOX2) were found only in a subset of cells in dermal spheres grown *in vitro*. We postulate that the hDPC colonies are composed of a heterogeneous population of cells some of which originate from hair follicle connective tissue sheath (Biernaskie et al., 2009; Rahmani et al., 2014) and others potentially from progenitors residing in the reticular dermis (Driskell et al., 2014; Schmidt and Horsley, 2013). Indeed, some hDPCs (or their progeny) may also be included in our hF preparations, which may explain overlapping benefits of hDPCs and hFs in the current study. Investigation into prospective markers to improve isolation and expansion efficiency of dermal progenitors will be of great importance when moving toward use of these cells for clinical applications. Nonetheless, both cell types readily proliferated in culture over several passages, providing adequate cell numbers to perform transplantation within our mouse xenograft model.

Importantly, this study employs a xenograft model to enable investigation of adjunct therapies aimed at improving outcomes for both humans and animals with significant skin wounds. In the current study, STSGs were considered as accepted when well vascularized, stable human epithelium was established as visualized both grossly and confirmed histologically by epithelial appearance as well as the expression of human nuclear antigen. Human STSGs integrated well within the host mouse tissue, and the grafts maintained their structure and integrity. Interestingly, in this series, STSGs did not form hypertrophic scars as has been previously described in human patients (Holavanahalli et al., 2010; Lavker, 1979), but instead maintained their characteristic textured appearance. As well, we did not observe the hypertrophic scar response that has been reported when using STSG xenografts (Momtazi et al., 2013), which may reflect a difference in grafting technique or aftercare between these studies. Thus, we conclude that this model provides an effective pre-clinical screening tool to investigate therapeutics to augment the success and function of a human STSG. Importantly, as this xenograft model utilizes a rodent lacking a full complement of immune cells, an autologous large animal model that more closely resembles that of human cutaneous anatomy may be useful to further investigate candidate therapeutics identified before a human clinical trial.

Transplanted human DPCs survived long term (up to 6 months, Figure S4), but importantly did not generate any aberrant growth and were never observed to stray far beyond the margins of the wound (Figure S3). Rather, after 3 months, hDPCs generated new dermal tissue and synthesized pro-collagen and other dermal ECM proteins that were not observed to the same degree in STSGs without cells. Markers consistent with a progenitor state, such as SOX2, were no longer expressed by the hDPCs following transplantation, and regionally distinct markers of interfollicular dermis were adopted supporting the hypothesis that donor cells are terminally differentiating in response to local cues within the wound environment.

The acellular collagen scaffold we utilized here (Primatrix, Integra) has been used clinically in prepared human wound beds to support granulation and revascularization of the wound bed before STSG (Kavros et al., 2014; Strong et al., 2016). Unsurprisingly, our results showed that addition of this particular scaffold at the time of grafting impeded graft take, since it may act as a barrier to revascularization of the STSG from the wound bed. However, the presence of the scaffold did yield superior results for hDPC engraftment and proliferation. Addition of the scaffold also increased dermal thickness, potentially allowing for greater volume for proliferation of hDPCs and ultimately leading to greater dermal tissue regeneration. Therefore, our results support the notion that a scaffold for transplantation of hDPCs would most likely improve dermal regeneration within STSG. We are currently developing flowable hydrogel scaffolds that impart adhesive properties to aid in graft application, as well as structural and mechanical support to maintain donor cell delivery, survival, and distribution beneath the graft without negatively affecting graft take. Our data and experience suggest that such a revised dermal matrix, when inoculated with hDPCs, could potentially support neodermal regeneration and re-epithelization in a single-staged procedure while avoiding the potential negative consequences observed with the particular scaffold here. These scaffolds are designed to be applied at the time of STSG rather than current dermal substitutes which are typically applied and allowed to vascularize for several days before STSG, which obligates the need for additional surgical procedures (Shahrokhi et al., 2014).

Immobility, pain, and chronic itch are challenges reported by patients living with skin grafts (Burnett et al., 2014; Holavanahalli et al., 2010). Epidermal function of

the grafted skin appeared to be maintained following grafting in all treatment groups. Interestingly, however, it was subjectively noted that many KI67+ cells were observed in the suprabasal layer of the epidermis in all grafted groups. Previous studies have identified an increased proportion of suprabasal KI67+ cells in inflammatory epidermal skin conditions such as psoriasis (Ando et al., 1990; Kim et al., 2018). As we did not co-stain with specific basal layer marker, we elected not to quantify the spatial relationship of these KI67+ cells between groups here. Nevertheless, as psoriatic lesions have been reported in human STSGs, this may be an important consideration for future studies. Importantly, a significant reduction in the incidence of severe pruritus was observed following the addition of cells. Both an increase in mast cell presence as well as abnormal re-innervation have been implicated as a possible cause of pruritus as well as contracture following wound healing (Monument et al., 2015). In grafts supplemented with an empty scaffold, diminished re-innervation and persistence of mast cells was observed. These negative effects were mitigated by the addition of hDPCs, highlighting an opportunity for further clinical improvement after the identification of a more permissive scaffold. Finally, we utilized an innovative, biaxial, analysis of mechanical properties of the grafts to best recapitulate the innate biomechanical function of skin on the patient. In doing so, we observed that the addition of cultured dermal cells improved the stiffness and stretch of the grafted areas more toward that of unwounded skin. A modest improvement of hDPCs over hFs was observed for maximum stiffness, suggesting that this population of cells may also yield better biomechanical outcomes for patients living with STSGs.

Taken together, we show that hDPCs are an accessible source of expandable cells for transplantation that readily engraft into the wound bed, generate new dermal tissue, support graft integration, and enhance several key functional aspects of the grafted skin. There are a number of important considerations that need to be addressed to advance translation of this approach. First, determination of markers to prospectively identify pure hDPCs will undoubtedly deliver the best results. Second, how donor age and body region from which hDPCs are cultured impact generation of sufficient numbers of functional hDPCs will also need to be determined. Third, the requirement of automated processes for rapid, reproducible expansion of cells will be critical to the development of any cell-based therapy (Surrao et al., 2016). Finally, our results show that the co-transplantation of hDPCs with a scaffold can significantly enhance their ability to engraft and proliferate within the wound. Efforts to engineer scaffolds that maintain viability and promote distribution of donor cells across the wound bed, without impeding graft take, will be paramount.

EXPERIMENTAL PROCEDURES

Cell Isolation and Culture

Adult human skin samples were obtained from deceased organ donors through the Southern Alberta Organ Transplantation and Tissue Recovery Program or from consenting patients undergoing plastic surgery in Calgary, Canada. All experiments involving animals and/or human skin samples received before approval from the Health Sciences Animal Care Committee and the Conjoint Health Ethics Review Board at the University of Calgary, respectively.

Cell Isolation

Adult (30–80 years old) human DPCs were isolated from thigh or scalp tissue according to previously published methods (Hagner and Biernaskie, 2013). Tissue was cleaned of underlying fat, minced, and incubated in Dispase (5 mg/mL) at 37°C for 3–4 h. Tissue was washed in Hank's balanced salt solution (HBSS) and the epidermis removed from the dermis using fine forceps. Remaining tissue was minced and then incubated in collagenase type XI (2×, Sigma) and DNase I (1×, Sigma) for 3–4 h, after which 1% fetal bovine serum (BioWhittaker) was added to inhibit enzymatic activity. Cells were triturated, diluted in HBSS and filtered through a 70 μm cell strainer (VWR) then plated in DMEM:F12 (3:1, Invitrogen) containing 1% (v/v) penicillin/streptomycin (BioWhittaker), 1 μg/mL Fungizone (Invitrogen), 2% (v/v) B27 supplement (Invitrogen), 40 ng/mL fibroblast growth factor 2 (FGF2) (10 μg/mL, BD Biosciences), and 25 ng/mL PDGF-BB (25 μg/mL, R&D Systems); hereafter referred to as Proliferation Medium (PM) (1×). Primary cultures were prophylactically treated with Plasmocin (0.02% [v/v], Invitrogen) and absence of *Mycoplasma* in all cultures was confirmed by PCR.

Cell Culture

Cells were cultured in approximately 10 mL PM per 25 cm^2 tissue-culture flasks (VWR) in a tissue-culture incubator at 37°C, 5% CO_2. Spherical colonies were allowed to grow for 7–21 days without passaging. The cultures were fed with 5× PM containing 10% (v/v) penicillin/streptomycin, 8 μg/mL Fungizone, 20% (v/v) B27, 200 ng/mL FGF2, and 80 ng/mL epidermal growth factor every 3 days. hDPCs were passaged using collagenase type IV and mechanical dissociation of spheres and splitting 1:2 with 50% fresh 1× PM and 50% filtered conditioned medium from the initial flask.

Immunocytochemistry

Cell suspensions each were loaded onto cytospin slides and centrifuged at 800 rpm for 5 min. Cells were fixed with 4% paraformaldehyde (PFA) at room temperature (RT) for 10 min. Following overnight permeabilization at 4°C with 0.5% Triton X-100/10% normal goat serum, cells were incubated with primary antibodies (diluted in 2% normal goat serum) overnight at 4°C. Following extensive washes, cells were exposed to secondary antibodies for 1 h at RT. Hoechst dye was added for 5 min to label nuclei, which were then washed and coverslipped with aqueous mounting medium. Primary and secondary antibodies used in this study can be found in Tables S1 and S2.

Lentiviral Transduction

hDPCs were transduced after three passages with high-titer lentiviral vectors expressing TdT (using the ubiquitous elongation

factor-1α [EF-1α] promoter). Spheres were dissociated to a single-cell suspension with collagenase type IV and transduced with 20 μL/mL lentivirus supernatant over 18 h in the presence of 8 μg/mL Polybrene in a tissue-culture incubator at 37°C, 5% CO_2. Cells were then flooded with 1× PM and cultured and passaged as described above. TdT+ cells were sorted on a FACSAria III (Becton Dickinson) and viable cells were detected using Fixable Viability Dye (eFlour 780, Thermo Fisher Scientific). Gates were set according to negative controls (non-transduced cells). Sorted cells were subsequently cultured and passaged as above.

Skin Grafting

STSG Harvest

FT human skin was collected from abdominal surgical waste tissue. Using a power-driven dermatome (Fixable Viability Dye, Integra, Padgett), split-thickness skin was harvested at a thickness of 0.015 inches. The skin was used immediately or stored in either RPMI-1640 or DMEM (Invitrogen) medium at 4°C for up to 7 days until grafting.

Mice

Skin grafts and cell transplants were performed in adult (6–12 weeks of age) male immune-compromised mice (Nu/Nu; Charles River).

hDPC Preparation

hDPC spheres were dissociated to single cells as above and resuspended at a density of 2×10^6 cells per 50 μL for 1.2 cm diameter circular wounds and 5×10^6 cells per 200 μL for 2 × 2 cm diameter circular wounds in a 5:1 mix of DMEM and Matrigel for injection without a scaffold and in DMEM alone when utilizing a scaffold.

Surgery

Animals were anesthetized with isoflurane (4% induction, 2% maintenance), eyes were lubricated with artificial tears gel (Tear-Gel, Alcon), and the skin on their dorsal back aseptically prepared with 70% ethanol and PBS. FT skin wounds were made on the dorsal back skin, either using a 1.2 cm diameter biopsy punch, or sterile scissors for 2 × 2 cm rectangular wounds. A rehydrated scaffold with or without cell suspension, cell suspension alone, or no treatment was placed into the wound bed and a human STSG was secured using 6-0 Vicryl suture in a simple interrupted pattern spaced. Grafts were dressed with Mepilex Ag followed by a circumferential abdominal bandage of gauze and Elastoplast. A 1 mL warm saline solution and a dose of buprenorphine (0.1 mg/kg) was administered following surgery and daily for up to 72 h after surgery. Dressings were replaced after 5–7 days and removed entirely after 21 days. Subsequently, an emollient cream (Glaxal Base) was applied daily to the graft.

Experimental Groups

Animals were separated into two control groups and three experimental groups. For the first control group (STSG), only an STSG was placed in the wound and sutured into place. The second control group (STSG/S) utilized an empty scaffold (Primatrix; Integra) underneath the graft alone. Before placement, this scaffold (4 × 4 cm fenestrated sheet, no. 607-004-440) was trimmed to fit the wound, rehydrated by submersion in sterile DMEM, and placed into the wound beneath the STSG. For the three experimental groups, resuspended hDPCs or hFs were injected under the STSG either with (STSG/S/hDPCs and STSG/S/hFs) or without a scaffold (STSG/hDPCs and STSG/hFs). Scaffold-alone groups received an equivalent amount of DMEM without cells. After identification of superior cell engraftment when delivered atop the scaffold, only two experimental groups (STSG/S/hDPCs and STSG/S/hFs) were used for all analyses beyond initial cell engraftment (Figure 2). To account for differences in skin donors, treatment and control grafts were completed in groups allowing direct comparison within the same human skin donor (n = 3). For some analyses, we also included hFT (uninjured) skin as a reference comparison.

Tissue Collection

Grafts were harvested at pre-determined time points. Mice were anesthetized with sodium pentobarbital (200 mg/kg intraperitoneally) and transcardially perfused with 15 mL of PBS (1×, Invitrogen), followed by 30 mL of 4% PFA. Grafts were excised and placed in 4% PFA overnight at 4°C, cryoprotected with 30% sucrose solution at 4°C, and then snap frozen in optimal cutting temperature compound (VWR). Grafts were sectioned at a thickness of 30 μm using a Leica CM1950 cryostat and stored at −80°C for immunohistochemical analysis.

Histology

Samples were stained using H&E staining and digitally captured using an Olympus virtual slide system macro slide scanner.

Graft Take Scoring/Calculations

Grafts were imaged at weeks 1, 2, 3, 8, and 12 weeks after transplant. Scoring of graft take was performed at 3 months by a blinded observer and assessed as either lost, partially accepted, or accepted, and quantified as percentages relative to all grafts performed.

Immunohistochemistry

All immunohistochemistry in grafted skin and control human skin was performed as follows. Sections were incubated with primary antibodies in PBS containing 0.05% Triton X-100 and 5% donkey serum overnight at 4°C. Secondary antibodies were incubated for 1 h at RT. Nuclei were stained with Hoechst for 10 min at RT. The slides were mounted using Permafluor mounting media. For all quantifications, 6 sections each acquired 500 μm apart for each animal (n = 4–5 per group) were immunostained, and images acquired using a Zeiss Observer microscope and scored or measured by a blinded observer.

Toluene Blue Staining and Quantification of Mast Cells

Mast cells were stained with toluidine blue working solution for 2 min. Slides were permanently mounted using a resinous mounting media following routine dehydration in alcohol and xylene.

Observation of Scratching following Grafting

Animals were observed daily for evidence of self-trauma due to scratching. At 3 months, observations were made by a blinded observer and animals were scored as mild, moderate, or severe scratching or none. If animals showed evidence of severe scratching they were euthanized and removed from the study.

In addition, animals were placed into glass chambers for observation of behavior and allowed to acclimatize for 10 min before being video-recorded for a period of 20 min. Videos were reviewed by a

blinded observed and scratching events were quantified for each animal.

Biaxial Analysis and Quantification

Tensile testing was performed on a four-motor planar biaxial system (ElectroForce Systems, TA Instruments, Springfield, MO).

Samples were cut into squares of 7 × 7 mm. Using a surgical skin marker, five dots were drawn on the center of the tissue, which was mounted to the four motors using four fish hooks on each side and silk sutures. The five dots were tracked during testing using a digital video extensometer camera. Samples were submerged in PBS (pH 7.4) kept at a 37°C.

The distance between hooks was measured for each sample in both directions, as was the sample thickness, to determine the undeformed dimensions of the tissue. Specimens were initially preloaded to 500 mN in both axes to eliminate sagging of the lines and tissue sample. An equibiaxial loading was applied to a strain of 60% in both undeformed directions. Samples were conditioned for each protocol by running the test for 10 cycles at a rate of 0.33 mm/s. Data were collected on the last or second to last cycle. The skin tissue was considered to be an incompressible, homogeneous, hyperelastic material undergoing finite quasistatic deformation.

Four deformation gradient tensors of the form

$$[\boldsymbol{F}] = \begin{bmatrix} \lambda_1 & \kappa_1 \\ \kappa_2 & \lambda_2 \end{bmatrix},$$

where λ_1 and λ_2 are the in plane stretches, and κ_1 and κ_2 are the in plane shear components, are obtained by tracking the displacement of the five dots and averaged. From the incompressibility assumption $J = \det(\mathbf{F}) = 1$ and λ_3 is found as

$$\lambda_3 = \frac{1}{\lambda_1 \lambda_2 - \kappa_1 \kappa_2}$$

The stress field of the specimen is estimated using the first Piola-Kirchhoff tensor $\mathbf{P}$. The only two non-zero components, in the direction of the loading axes, are $P_{11} = \frac{f_1}{L_2 h}$ and $P_{22} = \frac{f_2}{L_1 h}$, where f_1 and f_2 are the forces measured by the load cells, L_1 and L_2 are the undeformed lengths of the tissue, measured from hook to hook, and h is the undeformed thickness.

The second Piola-Kirchhoff stress, found as found as $\boldsymbol{S} = \boldsymbol{F}^{-1}\boldsymbol{P}$, versus the Green-Lagrange strain $\boldsymbol{E} = \frac{1}{2}(\boldsymbol{F}^{\boldsymbol{T}}\boldsymbol{F} - \boldsymbol{I})$, where $\boldsymbol{I}$ is the identity tensor, are plotted for both the loading and unloading portions of the cycle. The energy loss is found as the percentage ratio of the area of the tissue hysteresis (the difference between the loading and unloading curves) to the total area under the loading curve.

$$\text{Energy Loss} = \frac{\text{Load Area} - \text{Unload Area}}{\text{Load Area}} * 100.$$

For each sample, the tangent to the curve of first Piola-Kirchhoff versus local stretch λ is found at points from $\lambda = 1.00$ to λ_{max} at increments of 0.0025, from which a stiffness curve is generated. The maximum stiffness of the tissue is extracted as the maximum value.

Statistical Analysis

All statistical analysis was undertaken using GraphPad Prism (v.7.03). For comparisons between two groups, a standard t test was used. For comparisons between multiple groups, ANOVA tests were used with post hoc Turkey or Dunnett's multiple comparison tests. Significance was set at $p < 0.05$, and all graphs are represented as mean ± SEM. All p values can be found in Table S3.

SUPPLEMENTAL INFORMATION

Supplemental Information can be found online at https://doi.org/10.1016/j.stemcr.2019.10.011.

AUTHOR CONTRIBUTIONS

N.A.A., H.D.S., V.G., and J.B. conceived of the study and designed experiments. N.A.A., H.D.S., and J.B. conducted *in vivo* and *in vitro* studies, analyzed data, and wrote the manuscript. S.T. performed the biaxial studies, with support from N.L.R., who also conducted immunostaining. K.A. and G.Y. provided assistance with xenograft model development and data quantification. N.A.A. and H.D.S. prepared final figures for publication. D.N. and L.N.B. provided human tissue samples and technical guidance for xenograft model development. J.B., V.A.G., and E.S.D.M. supervised the project.

ACKNOWLEDGMENTS

The authors would like to thank the staff of the Southern Alberta Tissue Recovery and Transplant Program at Foothills Hospital, as well as members of the Section of Plastic Surgery, University of Calgary, for providing tissue samples. We also acknowledge Molly Chisholm, Martha Hurtatis, and Rodney Gamutin for outstanding animal care. This work was supported by the Stem Cell Network (to J.B. and V.A.G.), Canadian Institutes of Health Research, Alberta Innovates Health Solutions CRIO Team Grant, and the Calgary Firefighters Burn Treatment Society.

Received: October 25, 2018
Revised: October 15, 2019
Accepted: October 16, 2019
Published: November 14, 2019

REFERENCES

Abdelrahman, I., Elmasry, M., Olofsson, P., Steinvall, I., Fredrikson, M., and Sjoberg, F. (2017). Division of overall duration of stay into operative stay and postoperative stay improves the overall estimate as a measure of quality of outcome in burn care. PLoS One *12*, e0174579.

American Burn Association, 2016. National Burn Repository Report. Version 12.0.

Ando, M., Kawashima, T., Kobayashi, H., and Ohkawara, A. (1990). Immunohistochemical detection of proliferating cells in normal and psoriatic epidermis using Ki-67 monoclonal antibody. J. Dermatol. Sci. *1* (6), 441–446.

Auger, F.A., Berthod, F., Moulin, W., Pouliot, R., and Germain, L. (2004). Tissue-engineered skin substitutes: from in vitro constructs to in vivo applications. Biotechnol. Appl. Biochem. *39*, 263–275.

Biernaskie, J.A., McKenzie, I.A., Toma, J.G., and Miller, F.D. (2007). Isolation of skin-derived precursors (SKPs) and differentiation and enrichment of their Schwann cell progeny. Nat. Protoc. *1*, 2803–2812.

Biernaskie, J., Paris, M., Morozova, O., Fagan, B.M., Marra, M., Pevny, L., and Miller, F.D. (2009). SKPs derive from hair follicle precursors and exhibit properties of adult dermal stem. Cell Stem Cell. *5*, 610–623.

Burnett, L.N., Carr, E., Tapp, D., Raffin Bouchal, S., Horch, J.D., Biernaskie, J., and Gabriel, V. (2014). Patient experiences living with split thickness skin grafts. Burns *40*, 1097–1105.

Cantin-Warren, L., Guignard, R., Cortez Ghio, S., Larouche, D., Auger, F.A., and Germain, L. (2018). Specialized living wound dressing based on the self-assembly approach of tissue engineering. J. Funct. Biomater. *9*, 53.

Crandall, C.G., and Davis, S.L. (2010). Cutaneous vascular and sudomotor responses in human skin grafts. J. Appl. Physiol. *109*, 1524–1530.

Ding, J., and Tredget, E.E. (2017). Transplanting human skin grafts onto nude mice to model skin scars. Methods Mol. Biol. *1627*, 65–80.

Driskell, R.R., Jahoda, C.A.B., Chuong, C.-M., Watt, F.M., and Horsley, V. (2014). Defining dermal adipose tissue. Exp. Dermatol. *23*, 629–631.

Driskell, R.R., Lichtenberger, B.M., Hoste, E., Kretzschmar, K., Simons, B.D., Charalambous, M., Ferron, S.R., Herault, Y., Pavlovic, G., Ferguson-Smith, A.C., and Watt, F.M. (2013). Distinct fibroblast lineages determine dermal architecture in skin development and repair. Nature *504*, 277–281.

Engrav, L.H., Heimbach, D.M., Rivara, F.P., Kerr, K.F., Osler, T., Pham, T.N., Sharar, S.R., Esselman, P.C., Bulger, E.M., Carrougher, G.J., et al. (2013). Harborview burns—1974 to 2009. PLoS One *8*. https://doi.org/10.1371/journal.pone.0040086.

Fernandes, K.J.L., McKenzie, I.A., Mill, P., Smith, K.M., Akhavan, M., Barnabé-Heider, F., Biernaskie, J., Junek, A., Kobayashi, N.R., Toma, J.G., et al. (2004). A dermal niche for multipotent adult skin-derived precursor cells. Nat. Cell Biol. *6*, 1082–1093.

Gauvin, R., Larouche, D., Marcoux, H., Guignard, R., Auger, F.A., and Germain, L. (2013). Minimal contraction for tissue-engineered skin substitutes when matured at the air-liquid interface. J. Tissue Eng. Regener. Med. *7*, 452–460.

Hagner, A., and Biernaskie, J. (2013). Isolation and differentiation of hair follicle-derived dermal precursors. In Methods in Molecular Biology (Humana Press), pp. 247–263.

Holavanahalli, R.K., Helm, P.A., and Kowalske, K.J. (2010). Long-term outcomes in patients surviving large burns: the skin. J. Burn Care Res. *31*, 631–639.

Kim, S.A., Ryu, Y.W., Kwon, J.I., Choe, M.S., Jung, J.W., and Cho, J.W. (2018). Differential expression of cyclin D1, Ki-67, pRb, and p53 in psoriatic skin lesions and normal skin. Mol. Med. Rep. *17* (1), 735–742.

Kavros, S.J., Dutra, T., Gonzalez-Cruz, R., Liden, B., Marcus, B., McGuire, J., and Nazario-Guirau, L. (2014). The use of PriMatrix, a fetal bovine acellular dermal matrix, in healing chronic diabetic foot ulcers: a prospective multicenter study. Adv. Skin Wound Care *27*, 356–362.

Lavker, R.M. (1979). Structural alterations in exposed and unexposed aged skin. J. Invest. Dermatol. *73*, 59–66.

Lee, K.C., Joory, K., and Moiemen, N.S. (2014). History of burns: the past, present and the future. Burns Trauma *2* (4), 169–180.

Momtazi, M., Kwan, P., Ding, J., Anderson, C.C., Honardoust, D., Goekjian, S., and Tredget, E.E. (2013). A nude mouse model of hypertrophic scar shows morphologic and histologic characteristics of human hypertrophic scar. Wound Repair Regen. *21*, 77–87.

Monument, M.J., Hart, D.A., Salo, P.T., Befus, A.D., and Hildebrand, K.A. (2015). Neuroinflammatory mechanisms of connective tissue fibrosis: targeting neurogenic and mast cell contributions. Adv. Wound Care *4*, 137–151.

Nyame, T.T., Chiang, H.A., Leavitt, T., Ozambela, M., and Orgill, D.P. (2015). Tissue-engineered skin substitutes. Plast. Reconstr. Surg. *136*, 1379–1388.

Ong, Y.S., Samuel, M., and Song, C. (2006). Meta-analysis of early excision of burns. Burns *32*, 145–150.

Philippeos, C., Telerman, S.B., Oulès, B., Pisco, A.O., Shaw, T.J., Elgueta, R., Lombardi, G., Driskell, R.R., Soldin, M., Lynch, M.D., and Watt, F.M. (2018). Spatial and single-cell transcriptional profiling identifies functionally distinct human dermal fibroblast subpopulations. J. Invest. Dermatol. *138*, 811–825.

Rahmani, W., Abbasi, S., Hagner, A., Raharjo, E., Kumar, R., Hotta, A., Magness, S., Metzger, D., and Biernaskie, J. (2014). Hair follicle dermal stem cells regenerate the dermal sheath, repopulate the dermal papilla, and modulate hair type. Dev. Cell *31*, 543–558.

Schmidt, B.A., and Horsley, V. (2013). Intradermal adipocytes mediate fibroblast recruitment during skin wound healing. Development *140*, 1517–1527.

Shahrokhi, S., Arno, A., and Jeschke, M.G. (2014). The use of dermal substitutes in burn surgery: acute phase. Wound Repair Regen. *22* (1), 14–22.

Smolle, C., Cambiaso-Daniel, J., Forbes, A.A., Wurzer, P., Hundeshagen, G., Branski, L.K., Huss, F., and Kamolz, L.P. (2016). Recent trends in burn epidemiology worldwide: a systematic review. Burns *43* (2), 249–257.

Surrao, D.C., Boon, K., Borys, B., Sinha, S., Kumar, R., Biernaskie, J., and Kallos, M. (2016). Large-scale expansion of human skin-derived precursor cells (hSKPs) in stirred suspension bioreactors. Biotechnol. Bioeng. *113* (12), 2725–2738.

Strong, A.L., Bennett, D.K., Spreen, E.B., Adhvaryu, D.V., Littleton, J.C., and Mencer, E.J. (2016). Fetal bovine collagen matrix in the treatment of a full thickness burn wound: a case report with long-term follow-up. J. Burn Care Res. *37* (3), e292–e297.

Tiede, S., Kloepper, J.E., Ernst, N., Poeggeler, B., Kruse, C., and Paus, R. (2009). Nestin in human skin: exclusive expression in intramesenchymal skin compartments and regulation by leptin. J. Invest. Dermatol. *129*, 2711–2720.

Wang, J., Dodd, C., Shankowsky, H.A., Scott, P.G., and Tredget, E.E. (2008). Deep dermal fibroblasts contribute to hypertrophic scarring. Lab. Invest. *88*, 1278–1290.

Yi, J.W., and Kim, J.K. (2015). Prospective randomized comparison of scar appearances between cograft of acellular dermal matrix with autologous split-thickness skin and autologous split-thickness skin graft alone for full-thickness skin defects of the extremities. Plast. Reconstr. Surg. *135*, 609e–616e.

Stem Cell Reports, Volume *13*

Supplemental Information

Adult Human Dermal Progenitor Cell Transplantation Modulates the Functional Outcome of Split-Thickness Skin Xenografts

Natacha A. Agabalyan, Holly D. Sparks, Samar Tarraf, Nicole L. Rosin, Katie Anker, Grace Yoon, Lindsay N. Burnett, Duncan Nickerson, Elena S. Di Martino, Vincent A. Gabriel, and Jeff Biernaskie

SUPPLEMENTAL INFORMATION

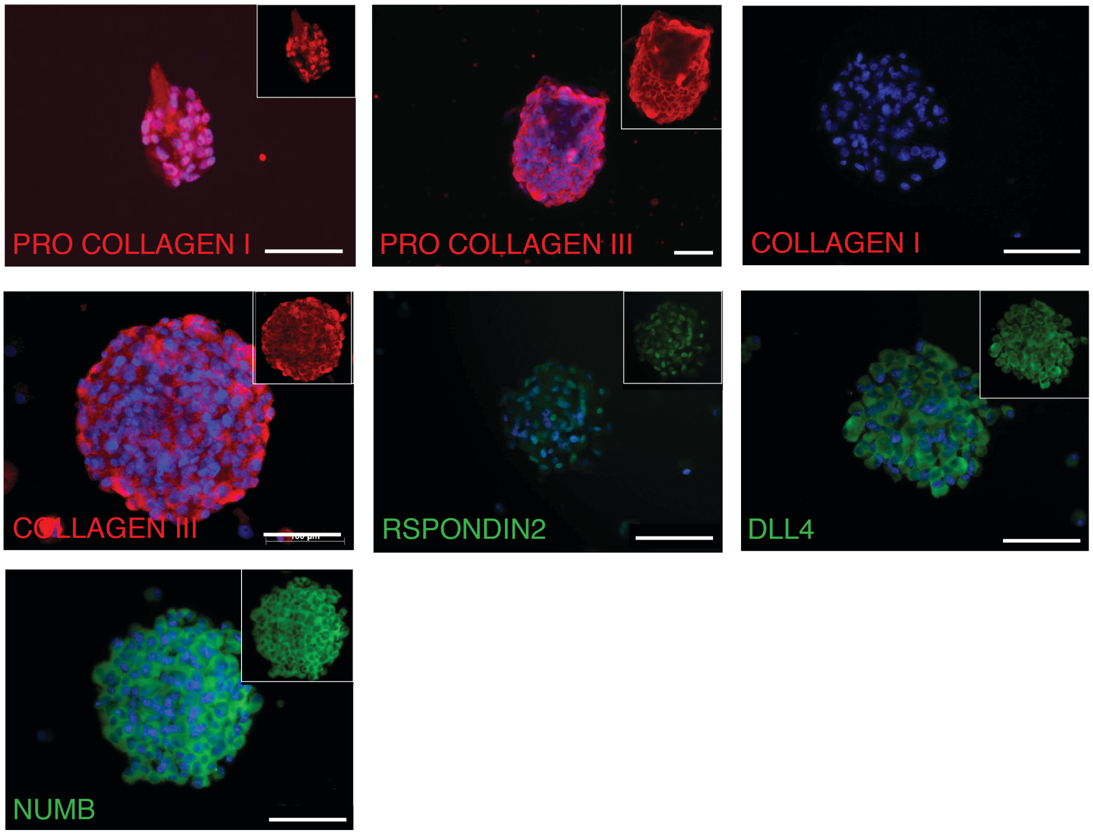

Figure S1. Extended immunocytochemical characterization of hDPCs *in vitro*. Several extracellular matrix proteins were expressed *in vitro*, including PRO COLLAGEN I and III and full-length COLLAGEN III, while full length COLLAGEN I was not. hDPCs in culture were found to consistently express DLL4, NUMB and RSPONDIN2. Scale bar = 100μm.

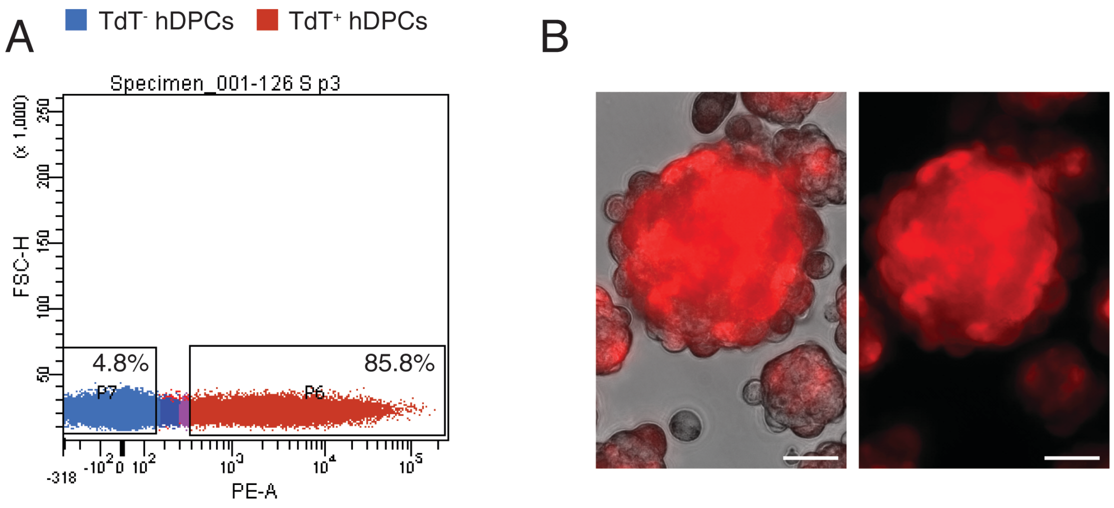

Figure S2. Transfection and FACS of hDPC cells with a TdTomato lentivirus (A) Fluorescence activated cell sorting strategy of TdT+ hDPCs. (B) Images of purified TdT+ hDPC culture following FACS. Scale bar = 100μm.

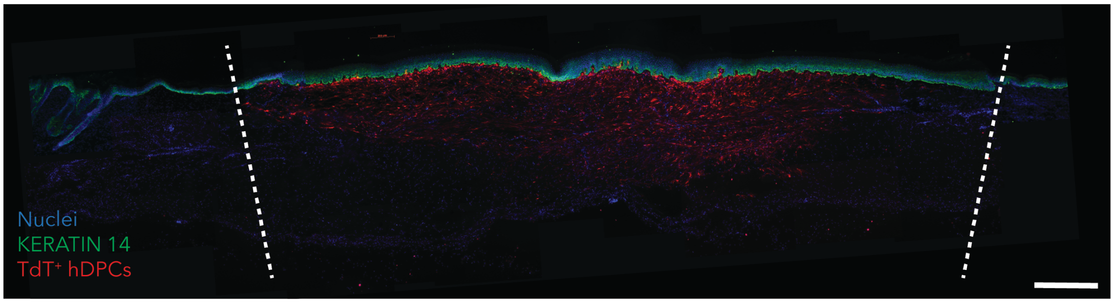

Figure S3. Transplanted TdT+ hDPCs respect the boundaries of the grafted area. Image shows engraftment of TdT+ hDPCs at 3 months *in vivo*. Dashed lines depict the boundaries of the xenograft, with intact mouse skin neighbouring. Labelled cells are not observed outside of the grafted area. Scale bar = 1cm.

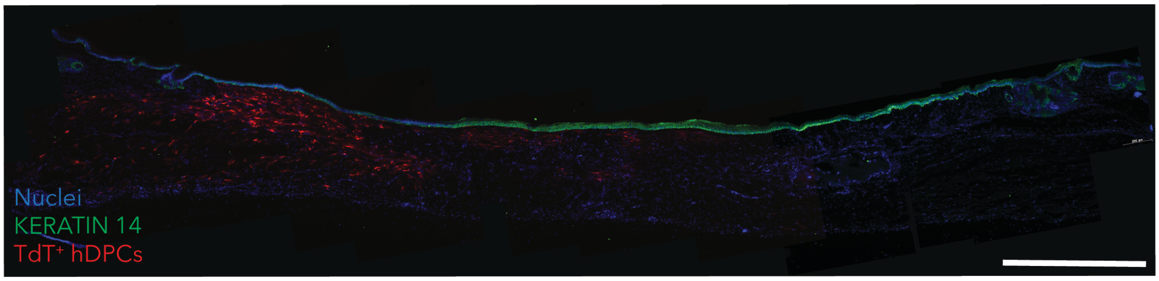

Figure S4. Transplanted TdT+ hDPCs were maintained in the graft for 6 months following transplantation. Image shows engraftment of TdT+ hDPCs *in vivo*. Scale bar = 1cm.

Table S1. Timing of transplants and harvests, clinical characteristics of skin sources

Human skin samples				
	Gender	Age	Anatomical location	Storage prior to harvest
**STSG #57**	Female	52 years old	Abdomen	-80°C in RPMI – thawed just prior to harvest
**STSG #58**	Female	52 years old	Abdomen	-80°C in RPMI – thawed just prior to harvest
**STSG #59**	Female	52 years old	Abdomen	-80°C in RPMI – thawed just prior to harvest
**STSG #60**	Female	N/A	Abdomen	Stored overnight in RPMI media at 4°C prior to harvest
**STSG #61**	Female	53 years old	Abdomen	Stored overnight in RPMI media at 4°C prior to harvest
**STSG #62**	Female	55 years old	Abdomen	Stored overnight in RPMI media at 4°C prior to harvest
**STSG #63**	Female	56 years old	Abdomen	Stored overnight in RPMI media at 4°C prior to harvest
**STSG #64**	Female	55 years old	Abdomen	Stored overnight in RPMI media at 4°C prior to harvest
**STSG #67**	Female	39 years old	Abdomen	-80°C in RPMI – thawed just prior to harvest
**STSG #70**	Female	36 years old	Abdomen	Stored overnight in RPMI media at 4°C prior to harvest
**STSG #71**	Female	36 years old	Abdomen	Stored overnight in RPMI media at 4°C prior to harvest
**STSG #72**	Female	55 years old	Abdomen	Stored overnight in RPMI media at 4°C prior to harvest
**STSG #153**	Female	34 years old	Abdomen	Stored overnight in RPMI media at 4°C prior to harvest

Table S2. Clinical characteristics of cell sources

Human DPC cell sources				
	Gender	Age	Anatomical location	Passage
**SATP #72**	Male	46 years old	Leg	P7, P8
**SATP #65**	Male	34 years old	Scalp	P5, P6
**SATP #82**	Female	78 years old	Scalp	P5
**SATP #90**	Female	53 years old	Scalp	P7
**SATP #122**	Female	46 years old	Scalp	P3
**SATP #126**	Male	66 years old	Scalp	P6, P7
**SATP #141**	Male	60 years old	Scalp	P6, P9
**Human Fibroblast cell sources**				
	Gender	Age	Anatomical location	Passage
**SATP #76**	Male	55 years old	Leg	P5
**SATP #90**	Female	53 years old	Scalp	P4
**SATP #122**	Female	46 years old	Leg	P8
**SATP #141**	Male	60 years old	Scalp	P10
**SATP #159**	Female	58 years old	Scalp	P6

Table S3. Primary antibodies used for immunocytochemistry and immunohistochemistry

Antigen	Supplier	Reference	Host	Concentration
**Collagen III**	Rockland	600-401-105S	Rabbit	1:100
**Fgf9**	Millipore	ABN41	Rabbit	1:100
**Fibronectin**	BD Biosciences	610078	Mouse	1:100
**Runx1-3**	Abcam	Ab92336	Mouse	1:50
**Sox2**	Stemgent	O9-0024	Rabbit	1:100
**PDGFR-α**	Santa Cruz	sc-338	Rabbit	1:100
**α-SMA**	Millipore	MABT381	Rabbit	1:100
**FSP1**	Millipore	07-2274	Rabbit	1:100
**Versican**	Millipore	AB1033	Rabbit	1:50
**Grem2**	Abcam	AB189387	Rabbit	1:200
**Pro Collagen I**	Millipore	ABT257	Rabbit	1:100
**Pro Collagen III**	Millipore	AB764P	Rabbit	1:100
**Biglycan**	Santa Cruz	sc-33788	Rabbit	1:100
**Notch1R**	Abcam	AB65297	Rabbit	1:200
**MCAM**	Millipore	04-1147	Rabbit	1:200
**Sox9**	Santa Cruz	sc-166505	Mouse	1:200
**Dll4**	Abcam	AB7280	Rabbit	1:200
**DLK1**	Abcam	AB21682	Rabbit	1:200
**Numb**	Abcam	AB14140	Rabbit	1:500
**Pax1**	ThermoFisher	PA5-51033	Rabbit	1:500
**Rspondin2**	R&D Systems	AF3266	Goat	1:500
**Loricrin**	Covance	PRB-145P	Rabbit	1:500
**Nestin**	Millipore	MAB5326	Mouse	1:100
**Collagen I**	Abcam	ab90395	Mouse	1:200
**Human Nuclear Antigen**	Millipore	MAB1281	Mouse	1:200
**Keratin 14**	Covance	PRB-155P	Rabbit	1:1000
**Ki67**	eBioscience	SolA15	Rat	1:100
**PGP9.5**	Thermofisher	PA5-29012	Rabbit	1:1000

Table S4. Secondary antibodies used for immunocytochemistry and immunohistochemistry

Antigen	Supplier	Reference	Host	Concentration
**Alexa-488**	Invitrogen	A-21206	Donkey anti-rabbit	1:500
**Alexa-488**	Invitrogen	SA1-72000	Donkey anti-chicken	1:500
**Alexa-555**	Invitrogen	A-31570	Donkey anti-mouse	1:500
**Alexa-555**	Invitrogen	A-21432	Donkey anti-goat	1:500
**Alexa-555**	Invitrogen	A-31572	Donkey anti-rabbit	1:500
**Alexa-555**	Invitrogen	A-11008	Goat anti-rabbit	1:500
**Alexa-546**	Invitrogen	A-21098	Donkey anti-sheep	1:500
**Alexa-647**	Invitrogen	A-21245	Goat anti-rabbit	1:500
**Alexa-647**	Invitrogen	A-21447	Donkey anti-goat	1:500
**Alexa-647**	Abcam	ab150155	Donkey anti-rat	1:500
**Hoechst**	Sigma-Aldrich	14530		1:1000
